# Novel Markers to Delineate Murine M1 and M2 Macrophages

**DOI:** 10.1371/journal.pone.0145342

**Published:** 2015-12-23

**Authors:** Kyle A. Jablonski, Stephanie A. Amici, Lindsay M. Webb, Juan de Dios Ruiz-Rosado, Phillip G. Popovich, Santiago Partida-Sanchez, Mireia Guerau-de-Arellano

**Affiliations:** 1 School of Health and Rehabilitation Sciences, Medical Laboratory Science Division, The Ohio State University, Columbus, Ohio, United States of America; 2 Biomedical Sciences Graduate Program, The Ohio State University, Columbus, Ohio, United States of America; 3 Center for Microbial Pathogenesis, The Research Institute at Nationwide Children’s Hospital, Columbus, Ohio, United States of America; 4 Department of Neuroscience, Wexner Medical Center at The Ohio State University, Columbus, Ohio, United States of America; University of Michigan Health System, UNITED STATES

## Abstract

Classically (M1) and alternatively activated (M2) macrophages exhibit distinct phenotypes and functions. It has been difficult to dissect macrophage phenotypes *in vivo*, where a spectrum of macrophage phenotypes exists, and also *in vitro*, where low or non-selective M2 marker protein expression is observed. To provide a foundation for the complexity of *in vivo* macrophage phenotypes, we performed a comprehensive analysis of the transcriptional signature of murine M0, M1 and M2 macrophages and identified genes common or exclusive to either subset. We validated by real-time PCR an M1-exclusive pattern of expression for CD38, G-protein coupled receptor 18 (Gpr18) and Formyl peptide receptor 2 (Fpr2) whereas Early growth response protein 2 (Egr2) and c-Myc were M2-exclusive. We further confirmed these data by flow cytometry and show that M1 and M2 macrophages can be distinguished by their relative expression of CD38 and Egr2. Egr2 labeled more M2 macrophages (~70%) than the canonical M2 macrophage marker Arginase-1, which labels 24% of M2 macrophages. Conversely, CD38 labeled most (71%) *in vitro* M1 macrophages. *In vivo*, a similar CD38^+^ population greatly increased after LPS exposure. Overall, this work defines exclusive and common M1 and M2 signatures and provides novel and improved tools to distinguish M1 and M2 murine macrophages.

## Introduction

Macrophages help maintain homeostasis during embryonic development and throughout life [1–4]. The diversity and overlap of cues in the microenvironment can generate a spectrum of *in vivo* macrophage phenotypes and functions [3,5–11]. The classical (M1) and alternative (M2) activation macrophage phenotypes are thought to be at the extremes of this spectrum [7]. Release of pathogen or danger-associated molecular patterns (PAMPs or DAMPs) and IFN-γ during infection or tissue injury promotes classical macrophage activation and ensures tissue sterility [5]. In contrast, IL-4 produced by Th2 lymphocytes during immune responses to parasitic infections or allergens alternatively activates macrophages, promoting wound healing and repair [12–14].

Efficient M1 macrophage responses are important for ensuring resistance to bacterial infection and are elicited by both gram-positive and gram-negative bacteria [5] to control pathogen growth [15–17]. Therefore, many pathogens such as *Salmonella*, *Brucella* and *Mycobacterium* have evolved mechanisms that interfere with M1 polarization [18–22]. Conversely, excessive or unresolved M1 macrophage activation can cause chronic inflammation and tissue damage [23]. Indeed, M1 macrophages have been implicated in the pathogenesis of several inflammatory conditions including atherosclerosis, diabetes and glomerulonephritis [4,14]. In the nervous system, M1 macrophages have been associated with multiple sclerosis, amyotrophic lateral sclerosis, stroke, spinal cord injury and traumatic brain injury [24–28]. A switch from M1 to M2 macrophages is thought to occur during natural resolution of inflammation and M2 macrophages are less toxic to microbes and vulnerable post-mitotic host cells (e.g., neurons). As a result, M2 macrophages are often described as having anti-inflammatory or reparative functions. However, like M1 macrophages, excessive or uncontrolled M2 macrophage activity can also cause diseases such as fibrosis or asthma [29].

The involvement of macrophage phenotypes in disease implies that detecting or modulating macrophage responses could be diagnostically and/or therapeutically useful. However, advances in the field have been hampered due to lack of consistent nomenclature, suboptimal M1/M2 macrophage phenotype markers, and differences between *in vivo* and *in vitro* macrophages. A new nomenclature [11], which indicates the stimuli used for macrophage activation [e.g., M(IL-10)], should clarify macrophage nature and allow comparison between studies; however, it may not be feasible to use this nomenclature to describe macrophages *in vivo* where the nature of the stimuli are complex and often are unknown. Difficulty detecting M1 and M2 macrophage phenotype *in vivo* is compounded by similar problems in *in vitro*-derived macrophages. For example, Arginase-1, which is a considered a classic M2 marker, is also up-regulated in M1 macrophages [30,31] and protein expression of Arginase-1 or CD206 is too low for reliable flow cytometry detection [3,5–11,32]. In conclusion, a more robust M1 vs. M2-discriminating system is critically needed to improve the detection and understanding of macrophage phenotype. *In vivo*, CD38 revealed an endotoxemia-induced macrophage population. Overall, this work provides gene signatures and tools to define macrophage phenotype.

Historically, M1/M2 marker discovery started in mouse macrophages using cDNA subtraction [7,30]. While this was followed by human macrophage transcriptome profiling [5,7,33–35], subsequent studies on mouse macrophages have deviated from clarifying shared and distinct M1/M2 signatures [12–14,36,37]. To provide a foundation for the complexity of *in vivo* macrophage phenotypes, we aimed to define the gene signatures associated with murine *in vitro* M1/M2 macrophage differentiation. Therefore, we performed transcriptional mRNA profiling in murine macrophages in either undifferentiated (M0), M1 or M2 conditions.

The profiling data indicate that, as a result of different activation stimuli, M1 and M2 macrophages co-express many genes, referred to as shared signatures. For example, the shared signature showed increases in transcription factors (TF) Kruppel-like factor (Klf) 4 and Activating Transcription Factor (Atf) 4. However, M1 and M2-specific gene signatures were identified as well. Among the top distinct genes in M1 or M2 macrophages, we validated CD38, Gpr18 and Fpr2 as novel M1 markers and Egr2 and c-Myc as M2 markers. A CD38/Egr2-based flow cytometry assay was capable of distinguishing M1 and M2 macrophages and provided an advantage over classic iNOS, Arginase-1 and CD206 phenotype markers. *In vivo*, CD38 revealed an endotoxemia-induced macrophage population. Overall, this work provides gene signatures and tools to define macrophage phenotype.

## Materials and Methods

### 2.1. Mice

Wild-type (WT) mice on the C57BL/6J background (Jackson Laboratories) were bred and kept in specific pathogen-free conditions at The Ohio State University Laboratory Animal Resources. All animal experiment procedures were approved under Ohio State University’s IACUC protocol # 2009A0036-R1 or #2013A00000151 to ensure the humane care and use of animals. Euthanasia was performed by cervical dislocation after ketamine/xylazine anesthesia or C02 treatment.

### 2.2. Bone marrow derived macrophages (BMDM)

To generate BMDM, the bone marrow cells from femurs and tibias from mice were harvested and cultured as previously described [5,28]. Briefly, isolated cells were incubated in Dulbecco’s Modified Eagle Media (DMEM, Mediatech, Herndon, VA) supplemented with 10% heat-inactivated fetal bovine serum (FBS) (Life Technologies, Grand Island, NY)), 1% penicillin/streptomycin, 1% glutamine, and 20% L929 cell supernatant (containing macrophage colony stimulating factor). On day 7 in culture the cells were washed, counted and replated in DMEM media (without L929 supernatant) at a density of 6-8x10^6^ cells/well (6-well plate, Falcon polystyrene). Cells were classically activated (M1 condition) with LPS (100 ng/ml, Sigma-Aldrich L2880) + IFN-γ (20ng/mL, eBioscience, San Diego, CA), alternatively activated (M2 condition) with IL-4 (20ng/mL, eBioscience) or received media alone (M0 condition). The LPS+ IFN-γ condition is used to simulate infectious and/or autoimmune conditions in which Th1 cells produce IFN-γ while pathogens or tissue damage provide PAMPs or DAMPs, respectively. Cells were harvested at the indicated time points, generally 24 hours post-stimulation, by washing in phosphate buffered saline (PBS) before cell lysis in miRVana Lysis buffer (Life Technologies) for total RNA isolation.

### 2.3. *In vivo* inflammation

For *in vivo* experiments, mice were injected with PBS or LPS (1 mg/kg, Sigma-Aldrich L2880). After 12 hours, mice were euthanized and spleen tissues were isolated from each mouse, minced with scissors and passed through a 40 μm cell strainer. To enrich macrophages, a Polymorphprep (Axis-Shield, Oslo, Norway) gradient was used according to manufacturer specifications. Briefly, 3 ml of cell suspension were layered on top of an equal volume of Polymorphprep and centrifuged for 30 minutes at 500 g (room temperature) without brake. The two top cellular layers containing monocytic and polymorphonuclear cells were recovered and washed in 10 mL PBS prior to staining as per flow cytometry protocol (see specific details for *in vivo* macrophage staining within flow cytometry section).

### 2.4. RNA Isolation

To examine RNA expression, cellular RNA was isolated using the miRVana kit (Life Technologies) according to manufacturer specifications. RNA quality/concentration was quantified using a Nanodrop spectrophotometer (ThermoScientific, Wilmington, DE) and/or Agilent bioanalyzer (Agilent Technologies, Santa Clara, CA). Samples were stored at -80°C until analysis.

### 2.5. Microarray

Total RNA was prepared from bone marrow-derived macrophages of WT mice (n = 2–3 independent mice) treated in M0, M1 or M2 conditions (n = 2–3 *in vitro* replicates per condition) for 24 hours using the miRVana isolation kit (Ambion). RNA quality was analyzed by the RNA 6000 Nano Chip (Agilent), and only samples with an RNA Integrity Number (RIN) >7 were used for further processing. RNA was processed and hybridized to the Affymetrix Mouse 430 2.0 chips at the Ohio State University Comprehensive Cancer Center (OSUCCC) Microarray facility. Raw data were normalized with the RMA algorithm implemented in the ‘‘Expression File Creator” module from the GenePattern software package [15–17,38]. Data were visualized with the Multiplot modules from GenePattern. Array data are deposited at the Gene Expression Omnibus (GEO) NCBI database with accession number (GSE69607), to become public upon manuscript publication.

### 2.6. Real-Time PCR

mRNA gene expression was determined using SYBR Green or Taqman quantitative Real-Time PCR on cDNA template. cDNA was generated from 500–1000 ng RNA per sample using oligo(dT)_12-18_ primers and Superscript III (Life Technologies), according to manufacturer’s instructions. Product was amplified with 0.5 μM forward and reverse primers of gene of interest and SybrGreen Mastermix (Life Technologies) or with commercially available Taqman primers and probe sets and Taqman Mastermix (Life Technologies) on an Applied Biosystems 7900 Real-Time PCR. The primer sequences for SybrGreen primer sets were the following: Fpr2 (mFpr2F 5’-TCTACCATCTCCAGAGTTCTGTGG-3’; mFpr2R 5’-TTACATCTACCACAATGTGAACTA-3’); mHprt-F (TGAAGAGCTACTGTAATGATCAGTCAAC) and mHprt-R (AGCAAGCTTGCAACCTTAACCA). The product numbers for ABI sets were the following: mCD38 (Mm01220906_M1), mGpr18 (Mm02620895), mC-myc (Mm00487804), mEgr2 (Mm00456650) and mβ-actin (Mm00607939_s1). Expression of target genes was normalized to β-actin/Hprt as a loading control. Real-Time PCR data was analyzed using the comparative Ct (ΔΔCT) method (ABI sets) or the standard curve method (mFpr2) depending on whether the test gene and β-actin gene amplification efficiencies were comparable or not, respectively [18–22,39,40].

### 2.7. Ingenuity Pathway Analysis

A gene list was compiled from the Affymetrix array results for Ingenuity Pathway Analysis (IPA) using the genes that were ≥2 fold change (FC) up-regulated in M1 vs. M0 but 2 FC down-regulated (<0.5FC) in M2 vs. M0 (M1-distinct genes) or genes that had a ≥2FC between M2 and M0 macrophages but a <0.5FC between M1 and M0 macrophages (M2-distinct genes). Additionally, genes that had ≥2FC of both M1 and M2 compared to M0 were analyzed (common genes).

### 2.8. Flow cytometry

BMDMs were differentiated for 24 hours in M0, M1 or M2 conditions and blocked with anti-mouse FcR antibody (CD16/CD32, BD) for 15 min at 4°C in FACS buffer (PBS with 2% FBS and 1 mM EDTA), subsequently cells were stained for 15 min at 4°C with blue-fluorescent reactive dye, L23105 (life technologies) to discriminate dead cells, and then surface stained with antibodies for CD11b (clone M1/70, V450 or PE, Biolegend) and CD38 (clone 90, FITC, eBioscience) or isotype control for 15 min at 4°C. Cells were washed three times with FACS buffer, fixed with the eBioscience Fixation/Permeabilization buffer for 40 min at 4°C and washed three times in 1X eBioscience Permeabilization buffer. For intracellular staining, cells were first blocked with anti-mouse FcR antibody (CD16/CD32, BD) in 1X Permeabilization buffer (eBioscience) for 45 min at 4°C prior to staining with anti-Egr2-APC antibody (clone erongr2, eBioscience), anti-Nos2-PE (CXNFT, eBioscience), anti-TNF-α-V650 (MP6-XT22, Biolegend) anti-Arg-1-APC (R and D systems), anti-CD206-Violet 650 (C068C2, Biolegend) or isotype control for 45 min at 4 degrees. After washing 3x in 1X Permeabilization buffer, cells were resuspended in FACS buffer and run on a BD FACSCanto II or BD LSRII Flow Cytometer (BD, NJ). Data were analyzed with FlowJo (Treestar, OR). For cytokine detection, macrophages were incubated in GolgiStop (BD) for 5 hours prior to staining.

For the M1/M2 discrimination flow cytometry experiment, BMDM cells were differentiated in M0, M1 or M2 conditions for 24 hours and harvested for flow cytometry. As controls, one set of M0, M1 and M2 macrophages were surface stained with CD11b-PE or CD11b-V450 and CD38-FITC and intracellular stained for Egr2-APC or isotype control. In addition, one set of M1 differentiated cells was stained with CD11b-V450 while a set of M2 cells was stained with CD11b-PE and washed three times. The pre-stained M1 and M2 cells were mixed at approximately 1.5:1 M1:M2 cell ratio and then underwent subsequent surface staining with CD38-FITC, fixation and intracellular Egr2-APC staining prior to flow cytometric analysis, as described above. The cell population was analyzed based on CD38/Egr2 staining and CD38^+^Egr2^-^ (candidate M1 markers) and CD38^-^Egr2^+^ (candidate M2 markers) gates were drawn. The percent of cells in each gate that originated from either the M1 or M2 culture was calculated based on the CD11b-V450 (M1 culture) or CD11b-PE (M2 culture) label.

For *in vivo* macrophage staining, the following dyes and antibodies were used: IndoA (LifeTech), Ly6G-PCPCy5.5 (1A8, Biolegend), CD11b-A700 (M1/70, Biolegend), F480-BV605 (BM8, Biolegend), Ly6C-e450 and CD38-FITC (clone 90, Biolegend). IndoA^-^Ly6G^-^CD11b^+^F480^+^ cells were gated for evaluation of % CD38^+^ cells and CD38 MFI. The CD38 MFI was also determined for the IndoA^-^Ly6G^-^CD11b^+^F480^+^ CD38^+^ population.

### 2.9. Statistical analysis

Statistical significance was determined using unpaired t-test (two-tail, equal SD) or analysis of variance (ANOVA) followed by Tukey post-hoc test. For microarray analysis, p values were Benjamin-Hochberg adjusted for multiple comparisons. Statistical significance was determined to be p<0.05. Analysis was completed using GraphPad Prism or GenePattern.

## Results

### 3.1. Defining M1 and M2 macrophage gene expression signatures

**Fig 1:**
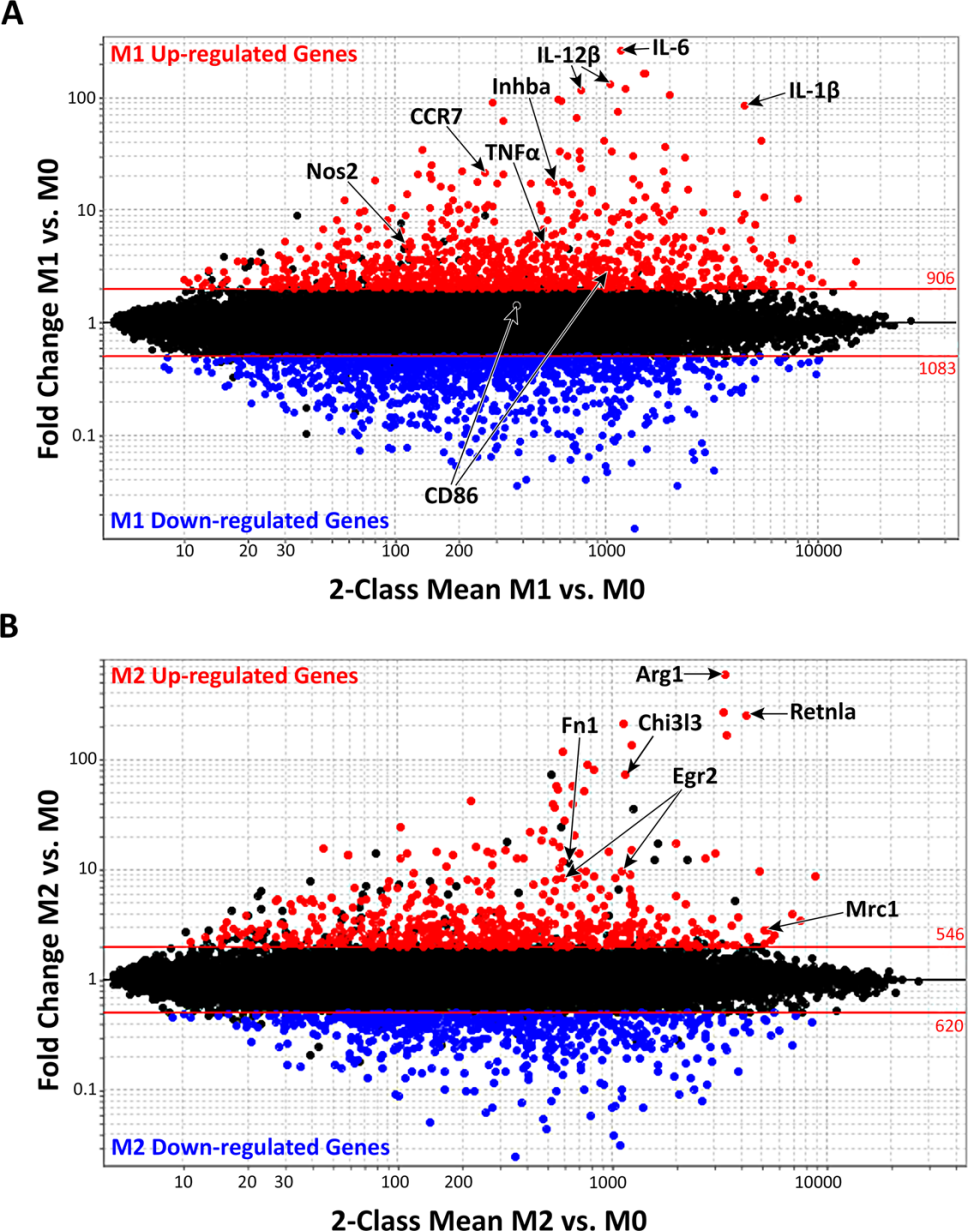
Macrophage signature in classically activated M1 and alternatively activated M2 macrophages. Fold Change (FC) vs. Mean Expression Value (MEV) plot of microarray data highlighting 2 FC or higher up-regulated genes (red, p≤0.05) or down-regulated genes (blue, p≤0.05) in **(A)** classically activated M1 macrophages (compared to M0 condition) or **(B)** alternatively activated M2 macrophages (compared to M0 condition). Red lines represent a ± 2FC cut-off. The numbers above and below the lines indicate the number of probes (some genes are represented by several probes and, hence, as several dots) above or below the 2 FC cut-off, respectively. Genes previously associated with classical and alternative macrophage phenotypes are labeled with corresponding gene names. Genes associated with (**A**) classically activated macrophages include Nos2, CCR7, TNFa, Inhba, IL12b, IL-6, and IL-1b, and CD86. Genes associated with (**B**) alternatively activated macrophages include Arg1, Fn1, Egr2 (human), Mrc1/CD206, Chi3l3/Ym1 and Retnla/Fizz-1.

Gene expression profiling was used to identify novel M1 and/or M2 macrophage gene signatures. Affymetrix M430 2.0 arrays were hybridized with complementary DNA (cDNA) from macrophages cultured in M0, M1 and M2 conditions. As shown in **Fig 1A**, more than one thousand genes, accounting for ~3% of the mouse genome, were differentially expressed between M1 and M0 macrophages. Specifically, the M1 macrophage “signature” was defined by increased expression (≥2FC) of 629 genes (p<0.05, red in **Fig 1A**, top 25 probes listed in **Table 1**) and decreased expression of 732 genes (≤0.5FC, p<0.05, blue in **Fig 1A**, top 25 probes listed in **Table 1**). Fewer genes, representing 1.8% of the mouse genome, were differentially expressed in M2 vs. M0 macrophages. The M2 macrophage signature was defined by 388 up-regulated genes (≥2 FC up, red in **Fig 1B**, top 25 probes listed in **Table 2**) and 425 down-regulated genes (≤0.5FC, p<0.05, blue in **Fig 1B**, top 25 probes listed in **Table 2**). To validate these data, we compared the current gene expression profiles with canonical M1 macrophage markers previously identified in mouse [23,41] or human macrophages [4,14,34]. Among these, 21 genes corresponding to canonical M1 markers such as Nos2, IL-1β, IL-6, IL-12β, CCR7, Inhba and TNF-α were also up-regulated in our M1 array (labeled in **Fig 1A**). Likewise, known M2 macrophage markers Arg1, Chi3l3/Ym1, Retnla/Fizz1, Egr2, Fn1 and Mrc1/CD206 were also expressed in our M2 dataset (labeled in **Fig 1B**). These data confirm those described in previous mouse or human M1 or M2 macrophage analyses and validate the robustness of our array results for more detailed data mining.

**Table 1:** Top 25 up-regulated or down-regulated gene probes in M1 vs. M0 macrophages.

M1 vs. M0					
Up-Regulated			Down-Regulated		
Gene	FC	p-value	Gene	FC	p-value
Il6	262.1	4.51E-02	Mrc1	0.0150	1.76E-03
Slc7a2	163.5	6.87E-03	S100a4	0.0360	3.01E-05
Slc7a2	163.0	8.01E-03	Ube2c	0.0360	2.96E-03
Il12b	130.0	1.16E-02	Slc9a9	0.0400	1.09E-03
Slc7a2	119.2	6.38E-03	Emp1	0.0410	1.96E-04
Il12b	114.7	1.49E-02	Slc40a1	0.0470	2.73E-03
Cxcl9	103.6	4.81E-04	Crip1	0.0480	5.28E-06
Serpinb2	96.5	3.70E-03	Slc40a1	0.0530	2.23E-02
Ptgs2	93.6	3.22E-02	Fam198b	0.0540	2.04E-05
Cxcl3	89.6	3.31E-02	Atp6v0d2	0.0570	1.20E-03
Il1b	85.5	2.52E-03	Ccnb2	0.0590	1.70E-03
Il1a	73.7	7.18E-03	St6gal1	0.0600	1.40E-03
Cd38	65.2	1.62E-02	Cd5l	0.0610	9.01E-03
Ptgs2	61.6	4.54E-02	Cbr2	0.0620	2.08E-04
Lcn2	41.8	1.74E-04	Clec10a	0.0640	7.48E-03
Ppap2a	41.0	3.63E-05	Atp6v0d2	0.0640	6.80E-03
Ptges	36.6	2.57E-03	Birc5	0.0650	5.81E-03
9130014G24Rik	34.6	3.02E-04	2810417H13Rik	0.0700	3.86E-03
Ptges	33.1	1.29E-02	Pparg	0.0700	3.35E-04
AA467197	32.9	4.81E-03	Trem2	0.0700	8.87E-05
Cd200	32.6	2.38E-03	Fcrl2	0.0700	1.43E-04
Ascl1	30.0	4.83E-04	Cd28	0.0710	5.13E-04
Traf1	30.0	2.52E-04	Slc13a3	0.0730	5.89E-04
Cd38	28.9	8.02E-04	Egr2	0.0740	6.71E-05
Ppap2a	28.1	8.74E-04	Rrm2	0.0750	7.74E-03

FC-Fold-change. Probe number and gene number may differ.

**Table 2:** Top 25 up-regulated or down-regulated gene probes in M2 vs. M0 macrophages.

M2 vs. M0					
Up-Regulated			Down-Regulated		
Gene	FC	p-value	Gene	FC	p-value
Arg1	582.7	6.15E-03	Ms4a4c	0.0249	8.41E-03
Mgl2	265.5	3.39E-07	Ifit3	0.0321	9.97E-03
Retnla	251.7	1.09E-04	Ifit2	0.0387	1.28E-02
Ear11	212.4	5.23E-03	Fpr2	0.0438	5.72E-03
Tmem26	166.7	1.93E-03	E030037K03Rik	0.0515	3.85E-03
Slc7a2	133.5	1.38E-03	Ifit1	0.0552	1.79E-02
Tmem26	115.3	3.11E-02	Ms4a4c	0.0580	6.60E-03
Socs2	89.6	1.73E-03	Gm14446	0.0617	1.66E-02
Ch25h	80.5	9.70E-05	Rsad2	0.0690	7.47E-03
Chi3l3	73.6	4.19E-04	Ms4a6b	0.0692	2.32E-03
Slcl7a2	57.8	1.02E-03	Slc40a1	0.0698	3.79E-02
Flt1	57.6	8.86E-04	Slc40a1	0.0725	7.65E-03
Slcl7a2	53.0	6.65E-03	Cmpk2	0.0760	4.76E-03
4833422F24Rik	50.9	8.46E-06	Rsad2	0.0791	5.15E-03
Socs2	41.4	5.43E-03	Tgtp2	0.0806	3.25E-03
Pdcd1lg2	39.5	3.88E-02	Rsad2	0.0852	1.72E-02
Chi3l4	39.4	5.45E-08	Emr4	0.0924	4.13E-02
Mcf2l	36.0	1.61E-03	Irf7	0.0930	3.57E-03
Ccl22	27.6	7.63E-05	Cxcl10	0.0967	1.45E-02
Cdh1	24.3	3.32E-04	Fpr1	0.0973	3.41E-03
Ccl17	22.7	1.97E-02	Ctla2b	0.0987	1.50E-03
Itgb3	21.9	1.29E-02	Slc1	0.0995	8.90E-04
AA467197	20.2	5.05E-03	Slc1	0.0999	2.39E-03
Il4i1	18.2	1.16E-02	Iigp1	0.1023	3.85E-03
Aqp9	17.6	4.89E-02	Fcgr1	0.1028	8.10E-04

FC-Fold-change. Probe number and gene number may differ.

### 3.2. Identification of common gene signatures in M1 and M2 macrophages

**Fig 2:**
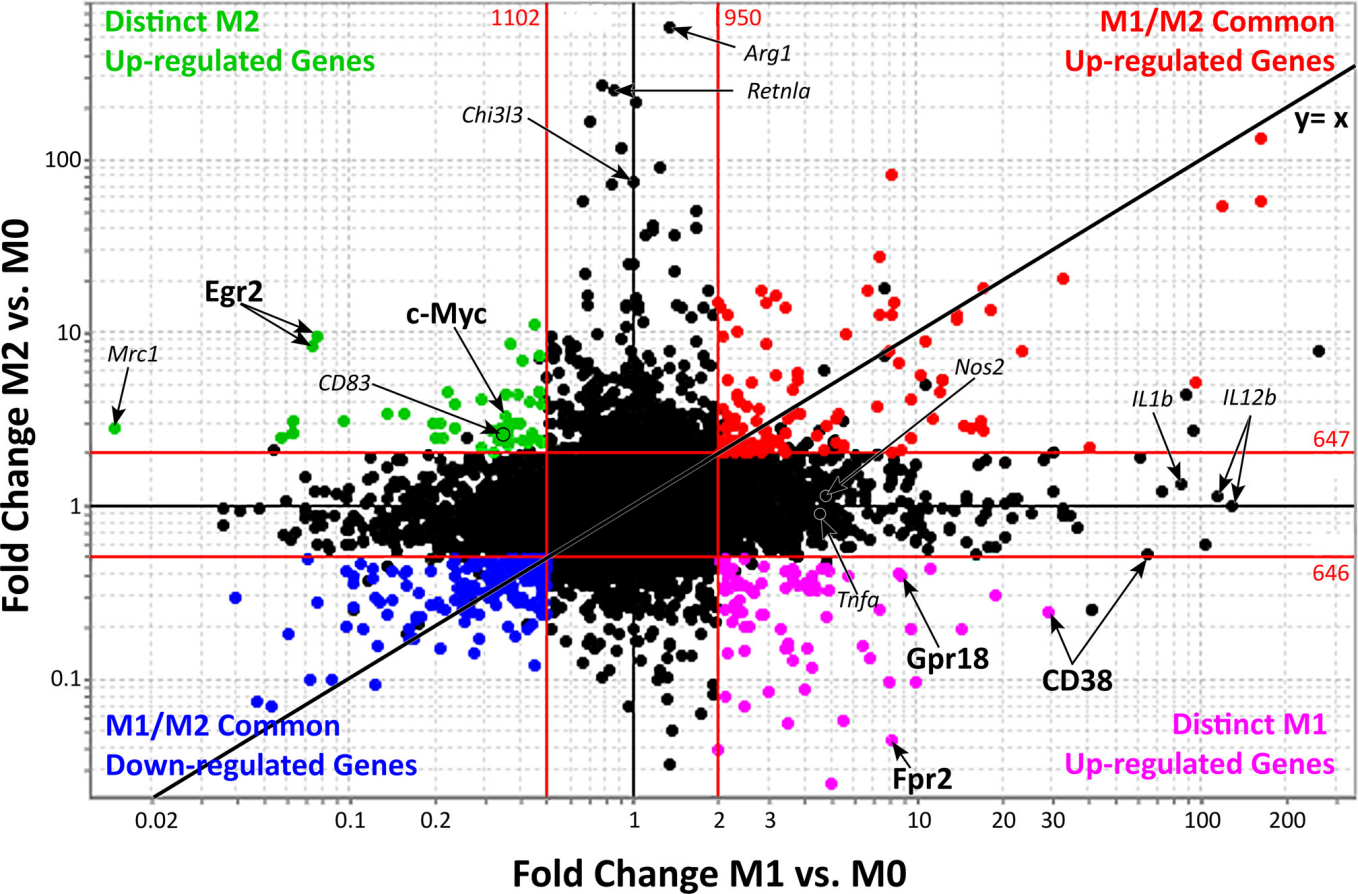
Comparison of common and distinct genes of classically activated and alternatively activated macrophages. Fold-change (FC) vs. FC plot of M1 vs. M0 on the x-axis and M2 vs. M0 on the y-axis highlighting common up-regulated genes in red (81 genes, 105 probes), common down-regulated genes in blue (125 genes, 172 probes), M1 up- and M2 down-regulated genes in purple (57 genes, 78 probes) and M2 up- and M1 down-regulated genes in green (33 genes, 45 probes). Arrows indicate distinct M1 or M2 genes. Red lines represent a ±2FC cut-off. The black line indicates the *x = y* diagonal expected if all gene probes were up- or down-regulated to the same extent in M1 and M2 macrophages (FC M1 vs. M0 = FC M2 vs. M0). Previously reported markers of classical and alternative macrophages are included for reference in small, italicized font. Genes representing classical macrophages include Nos2, Tnfa, IL-1b and IL12b. Genes representing alternative macrophages include Arg1, Mrc1 (CD206), Retnla (Fizz-1), and Chi3l3 (Ym1). The genes labeled in larger bold font, CD38, Fpr2 and Gpr18 or c-Myc and Egr2 were selected as candidate M1- or M2-selective markers, respectively.

M0 macrophages are presumed naïve cells that have not been stimulated or received signals that promote activation and functional polarization. Once M0 macrophages receive M1 or M2-specific signals, the resulting macrophages are termed "activated". To identify expressed genes that are common to activated M1 and M2 macrophages, gene expression values were plotted onto a FC vs. FC plot (**Fig 2**). In this type of plot, genes that are up-regulated in both M1 and M2 macrophages are found in the upper right quadrant while genes that are down-regulated in both are in the bottom left quadrant. In contrast, genes falling in the remaining two quadrants are exclusive to either M1 or M2 macrophages. Using the 2 and 0.5 FC lines as a reference, we identified 81 co-up-regulated (**Fig 2**, red, top 10 genes listed in **Table 3**, all probes listed in **S1 Table**) and 125 co-down-regulated (**Fig 2**, blue, top 10 genes listed in **Table 3**, all probes listed in **S2 Table**) genes. The “shared” M1/M2 macrophage signature represents 15–25% of individual M1 or M2 signatures. Among shared genes, a subset of TFs, metabolic enzymes and transmembrane proteins, appeared to be linked, as revealed by Ingenuity Pathway Analysis (IPA). Transcription factors Klf4, Atf4, Nuclear factor Interleukin 3-regulated (Nfil3), Human Immunodeficiency Virus Type I Enhancer Binding Protein (Hivep) 3 and Basic Helix Loop Helix (Bhlhe) 40 were up-regulated and linked to decreased expression of another set of transcriptional regulators, including Klf2, Transcription Factor Maf, Transcription Factor (Tcf) 4, Nuclear factor of activated T cells cytoplasmic (Nfatc) 2 and Regulator of calcineurin (Rcan) 1 (**S1 Fig**, bolded). These transcriptional networks control expression of genes that function as enzymes, transmembrane receptors, transporters, kinases, G-protein coupled receptors, peptidases, phosphatases and cytokines (**S3 Table**).

**Table 3:** Top 10 up- and down-Regulated genes common to M1 and M2 macrophages.

**Up-Regulated**	**M1 vs. M0**		**M2 vs. M0**	
**Gene**	**FC**	**p-value**	**FC**	**p-value**
Slc7a2	163.5	6.87E-03	133.5	1.38E-03
Serpinb2	96.5	3.70E-03	5.1	1.33E-03
Ppap2a	41.0	3.63E-05	2.2	1.96E-03
AA467197	32.9	4.81E-03	20.2	5.05E-03
Slc7a11	23.7	1.91E-02	7.7	2.70E-02
Al504432	17.2	2.02E-03	2.7	3.80E-04
Il4i1	17.2	6.78E-03	18.2	1.16E-02
Cd40	16.9	1.63E-04	3.1	1.46E-02
F10	14.0	3.28E-04	11.9	1.16E-02
Rasgrp1	12.3	3.53E-03	5.3	1.71E-02
**Down-Regulated**	**M1 vs. M0**		**M2 vs. M0**	
**Gene**	**FC**	**p-value**	**FC**	**p-value**
Sh2d3c	0.39	4.24E-04	0.48	3.73E-03
Slc13a3	0.07	5.89E-04	0.10	2.39E-03
Rcan1	0.40	2.25E-03	0.48	1.07E-02
4632428N05Rik	0.47	6.68E-05	0.30	1.53E-04
Trp53inp1	0.49	1.52E-02	0.47	7.32E-03
Nr1d2	0.43	4.70E-05	0.46	1.80E-04
Fcgrt	0.33	5.87E-03	0.43	2.63E-02
Slc40a1	0.05	2.73E-03	0.07	7.65E-03
Nfxl1	0.49	1.69E-02	0.24	9.84E-03
Il16	0.42	5.79E-03	0.44	9.82E-03

FC-Fold-change. Probe number and gene number may differ.

### 3.3. Distinct signatures of classically and alternatively activated macrophages

Besides shared genes, the FC vs. FC plot identifies genes that are differentially expressed in M1 or M2 macrophages (**Fig 2)**. To identify M1/M2-discriminating markers, we focused on genes up-regulated in one condition but down-regulated in the opposite condition. This strategy excludes non-selective genes, such as Arg-1, that are very highly up-regulated in M2 but are still up-regulated in M1 cells. Such analysis revealed 57 genes up-regulated in M1 macrophages, which were reciprocally down-regulated in M2 macrophages (bottom right quadrant, in purple, **Table 4**). These genes will be subsequently referred to as M1-distinct genes. Likewise, 33 genes were up-regulated in M2 macrophages but down-regulated in M1 macrophages (upper left quadrant, in green, **Table 5**). These genes will be referred to as M2-distinct genes. These two sets of genes provide a promising group of candidate M1 and M2 macrophage specific markers that may be used to distinguish between these uniquely activated macrophage phenotypes.

**Table 4:** Genes increased more than 2-FC in M1 and decreased more than 2-FC in M2 macrophages.

	M1 vs. M0		M2 vs. M0	
Gene	FC	p-value	FC	p-value
Cd38	28.87	8.02E-04	0.24	6.31E-03
Cfb	18.92	4.20E-04	0.30	3.29E-02
Slfn4	14.40	7.69E-03	0.19	2.29E-04
H2-Q6	11.25	5.94E-03	0.44	1.30E-02
Fpr1	9.97	8.40E-04	0.10	3.41E-03
Slfn1	9.46	1.66E-04	0.20	1.79E-02
Gpr18	8.78	1.15E-04	0.39	1.10E-02
Ccrl2	8.58	6.02E-03	0.41	8.07E-03
Fpr2	8.23	2.00E-05	0.04	5.72E-03
Cxcl10	7.95	2.13E-04	0.10	1.45E-02
Mpa2l	7.38	1.19E-03	0.25	5.50E-03
Mpa2l	6.80	1.06E-03	0.13	8.11E-03
Oasl1	6.47	1.79E-04	0.16	3.32E-02
Tlr2	5.71	1.74E-03	0.39	6.69E-03
Ms4a4c	5.47	4.61E-05	0.06	6.60E-03
Ms4a4c	4.98	9.04E-04	0.03	8.41E-03
LOC100503664	4.94	1.01E-04	0.33	6.87E-04
Irak3	4.89	1.46E-05	0.42	1.25E-02
Irak3	4.85	2.75E-04	0.44	1.59E-03
Hp	4.79	5.23E-04	0.23	4.36E-02
Itgal	4.63	5.17E-04	0.43	1.19E-02
Herc6	4.45	1.98E-03	0.33	4.90E-02
Herc6	4.37	2.21E-04	0.35	1.08E-02
Cd300lf	4.31	2.74E-03	0.40	1.41E-02
Isf20	4.26	5.89E-04	0.12	1.11E-02
Pstpip2	4.17	7.79E-06	0.33	9.87E-04
Cp	4.12	4.21E-03	0.34	4.34E-02
Isg15	4.11	1.55E-04	0.15	1.68E-02
Herc6	4.05	3.64E-03	0.36	4.39E-02
Probe 1452408_at	4.03	4.90E-04	0.09	1.74E-03
Cp	3.80	2.47E-03	0.35	6.16E-03
Cp	3.74	8.27E-04	0.41	8.82E-03
Ifi44	3.68	6.07E-03	0.13	6.81E-03
Pstpip2	3.67	5.55E-04	0.43	9.84E-03
Cp	3.65	1.02E-03	0.38	5.33E-03
E030037K03Rik	3.54	4.68E-03	0.16	8.31E-03
Saa3	3.50	5.83E-05	0.15	8.31E-03
Ifit1	3.48	5.03E-04	0.06	1.79E-02
Cp	3.46	5.25E-03	0.33	9.09E-03
Marco	3.44	2.70E-03	0.15	9.22E-04
F11r	3.43	1.98E-02	0.41	9.84E-03
Marco	3.33	2.37E-03	0.20	2.49E-03
Rsad2	3.03	1.75E-03	0.09	1.72E-02
Ddx60	2.89	6.52E-04	0.23	1.11E-02
Pilr1	2.89	1.22E-04	0.44	1.39E-02
Cpd	2.85	9.18E-03	0.36	1.18E-02
Fam26f	2.82	5.24E-03	0.23	2.95E-03
Aoah	2.65	6.33E-03	0.31	2.14E-03
Cpd	2.56	1.67E-03	0.34	2.17E-03
Gngt2	2.55	4.37E-04	0.20	2.96E-03
Mx1	2.49	1.46E-02	0.15	1.04E-02
Pyhin1	2.48	5.26E-03	0.20	7.00E-03
Cpd	2.48	9.04E-04	0.34	1.57E-03
Rsad2	2.47	9.79E-04	0.07	7.47E-03
Epb4.1l3	2.47	1.89E-02	0.50	2.07E-02
Slfn8	2.42	1.28E-04	0.24	2.48E-03
Arhgap24	2.38	6.48E-04	0.36	2.53E-03
Ddx60	2.36	7.01E-03	0.29	4.74E-03
Nfkbiz	2.34	2.68E-02	0.34	8.91E-04
Gbp6	2.31	2.54E-03	0.37	2.54E-02
Stat1	2.27	1.92E-03	0.27	2.27E-02
Zpb1	2.27	1.22E-03	0.25	9.01E-03
Cpd	2.27	2.77E-03	0.27	8.28E-04
D14Erd668e	2.22	2.74E-03	0.21	3.41E-03
Ddx58	2.22	7.19E-03	0.43	2.46E-02
Tuba4a	2.18	3.43E-03	0.33	1.07E-02
Tuba4a	2.14	1.30E-03	0.38	4.28E-03
Nfkbiz	2.14	4.04E-03	0.14	1.24E-03
H2-T10	2.14	2.67E-03	0.44	4.41E-03
Ebi3	2.13	1.10E-02	0.33	3.28E-04
Rsad2	2.12	1.71E-03	0.08	5.15E-03
Stat1	2.11	5.28E-03	0.34	3.41E-02
Fam176b	2.11	2.06E-03	0.49	5.71E-03
Xaf1	2.09	8.74E-04	0.25	8.04E-04
Gbp6	2.08	2.72E-03	0.44	2.13E-02
Stat2	2.08	2.93E-03	0.45	3.12E-02
Sepx1	2.01	3.29E-04	0.47	8.53E-03
Ifit2	2.01	9.28E-03	0.04	1.28E-02

FC = Fold Change. Probe number and gene number may differ

**Table 5:** Genes increased more than 2-FC in M2 and decreased more than 2-FC in M1 macrophages.

	M1 vs. M0		M2 vs. M0	
Gene	FC	p-value	FC	p-value
Ptgs1	0.449	5.20E-03	11.022	5.11E-05
Egr2	0.077	2.30E-03	9.469	1.59E-04
Olfm1	0.372	1.06E-03	8.456	1.20E-02
Egr2	0.074	6.71E-05	8.356	1.16E-04
Flrt2	0.472	1.26E-02	7.294	1.02E-04
Flrt2	0.405	4.47E-03	6.928	6.05E-05
P2ry1	0.472	3.53E-03	4.519	4.06E-03
Vwf	0.222	8.02E-03	4.512	1.99E-04
Bcar3	0.358	5.61E-04	4.429	2.68E-03
Il6st	0.395	2.22E-05	4.352	8.66E-04
Il6st	0.293	4.05E-04	4.03	2.71E-03
Tanc2	0.436	2.55E-05	3.946	2.41E-05
Mmp12	0.234	4.22E-04	3.887	7.31E-05
Tcfec	0.474	1.76E-04	3.794	1.19E-02
Clec7a	0.136	3.96E-05	3.398	1.54E-05
Matk	0.156	3.95E-07	3.386	4.21E-03
Myc	0.358	2.80E-03	3.254	4.87E-03
Clec10a	0.064	7.48E-03	3.092	2.91E-03
Matk	0.095	1.01E-03	3.066	4.57E-03
Amz1	0.397	5.34E-03	3.008	4.09E-04
Tmem158	0.368	4.16E-04	2.988	1.43E-02
Tanc2	0.385	1.61E-04	2.977	9.43E-03
Tiam1	0.196	8.39E-05	2.974	6.66E-05
Rhoj	0.208	6.56E-03	2.964	2.92E-02
Mmp9	0.235	4.98E-05	2.819	4.07E-03
Mrc1	0.015	1.76E-03	2.817	1.62E-03
Atp6v0a1	0.345	1.04E-02	2.815	3.36E-03
Atp6v0a1	0.318	6.87E-05	2.736	7.92E-04
Lmna	0.336	8.41E-05	2.736	1.44E-02
Chst7	0.36	1.66E-05	2.67	1.01E-02
Atp6v0d2	0.064	6.80E-03	2.601	9.36E-03
Gnb4	0.444	1.12E-03	2.576	1.66E-02
Atp6v0d2	0.057	1.20E-03	2.465	6.55E-03
Emp2	0.212	1.83E-04	2.459	2.31E-03
Cd300ld	0.202	9.37E-06	2.437	2.76E-04
Cd83	0.333	7.46E-04	2.381	1.32E-03
Socs6	0.367	7.54E-03	2.373	8.12E-03
Actn1	0.464	1.54E-04	2.366	2.20E-03
Socs6	0.407	9.16E-03	2.356	2.37E-03
Emp2	0.435	7.35E-03	2.355	1.65E-02
Socs6	0.428	3.42E-03	2.285	2.30E-03
Emp2	0.475	6.29E-03	2.26	2.82E-02
Atp6v0a1	0.361	2.22E-02	2.232	2.19E-02
Plk2	0.29	2.31E-04	2.151	3.28E-03
Ptpla	0.323	4.97E-03	2.041	1.49E-02

FC = Fold Change. Probe number and gene number may differ

To identify networks linking these genes, we performed IPA analysis on M1-distinct genes. This analysis revealed Stat1, Stat2 and Pyhin, a microbial sensor that drives inflammasome activation [24–28,42], downstream of LPS+IFN-γ-stimulated TLR signaling (**S2 Fig**). Stat activity has been linked to increased expression of Dead Box Polypeptide (Ddx) 58 [29,43], a putative helicase implicated in RNA binding. Ddx58 was in turn linked to many expressed M1 genes, including the secreted M1 marker Cxcl10. LPS stimulation was also linked to increased expression of transmembrane receptors Tlr2, Fpr2, Gpr18 and CD38. Among these M1 marker candidate genes, CD38, Fpr2 and Gpr18 (bold letter-labeled in **Fig 2,** other common M1 markers shown in italics for comparison) were among the top 10 in FC increase. Fpr2 and Gpr18 are G-protein coupled receptors and CD38 is a multifunctional ectoenzyme that synthesizes ADP-ribose and cyclic ADP-ribose and promotes intracellular Ca^2+^ signaling. Similar IPA analysis on M2-exclusive genes shows an IL-4 driven network that leads to increased expression of c-Myc and Egr2, two transcriptional regulators that are linked to each other and to other M2-exclusive candidate markers (**S3 Fig**). C-myc has important roles in cell cycle and metabolism [44] and Egr2 is known to have an inactivating role in T cells [45]. Egr2 was one of the most highly up-regulated genes in the M2-exclusive quadrant, among which we also found the well-known surface M2 markers Mrc1 and CD83 (labeled in **Fig 2**).

### 3.4. Validation of CD38, Fpr2 and Gpr18 as distinct M1 macrophage markers

**Fig 3:**
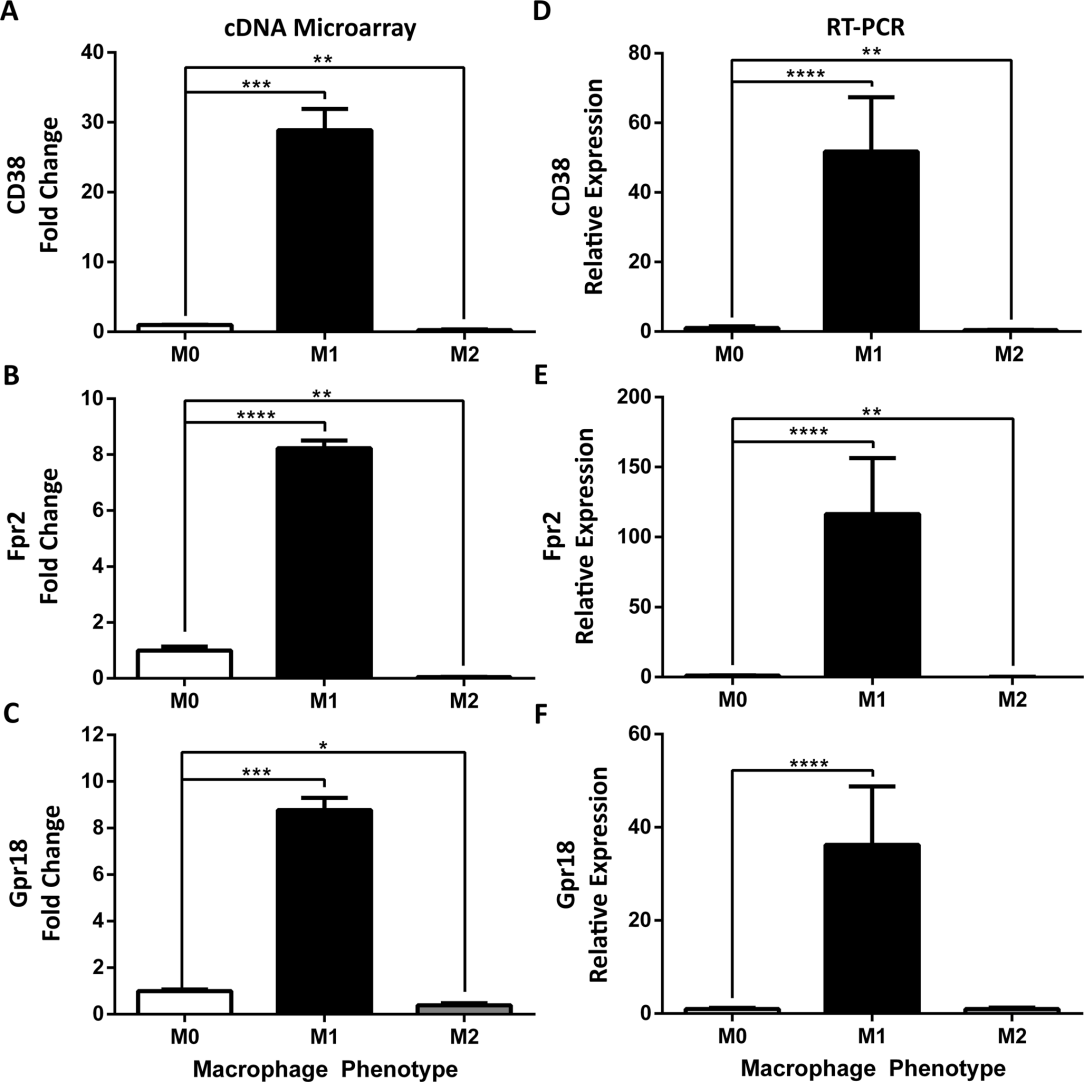
Identification and validation of novel classically activated M1 markers CD38, Fpr2 and Gpr18. Expression of (**A**) CD38, (**B**) Formyl peptide receptor 2 (Fpr2) and (**C**) G-Protein Coupled Receptor 18 (Gpr18) was determined by cDNA microarray in BMDMs stimulated for 24 hours in M0, M1, and M2 (n = 2–3 mice, 3 replicates/mouse/condition) conditions. Gene expression is represented as fold change relative to unstimulated M0 condition (FC ± SEM), multiple corrected t-test. Expression of (**D**) CD38, (**E)** Fpr2, (**F**) and Gpr18 was determined by Real-Time PCR in BMDMs stimulated for 24 hours in M0, M1, and M2 (n = 8, 6 mice, 1–2 replicates/mouse/condition) conditions. Gene expression expressed as fold-change relative to unstimulated M0 condition (FC ± SD); ANOVA followed by multiple comparison post-hoc t-test; *p<0.05, **p<0.01, ***p<0.001, ****p<0.0001.

To unequivocally discriminate between polarized macrophage subsets (e.g., M1 or M2), we focused on distinct M1 up-regulated genes highlighted in purple in **Fig 2**. Among the top 10 genes in this group (**Table 4**), we focused on CD38, Fpr2, and Gpr18 as M1 marker candidates due to their predicted membrane expression patterns that could lead to improved M1 detection and sorting by flow cytometry. These candidate markers were verified by Real-Time PCR on independent datasets. CD38 was the most highly and significantly up-regulated in M1 vs. M0 macrophages, with close to a 30-fold increase in M1 macrophages (**Fig 3A**). Fpr2 and Gpr18 were up-regulated 8-fold (**Fig 3B and 3C**). All three genes were down-regulated 2- to 25-fold in M2 macrophages (**Fig 3A–3C**), and displayed raw mean expression values <65 units, indicative of low-level expression in M2 macrophages.

To validate these array-identified genes as M1 markers, we performed Real-Time PCR on two independent datasets. CD38 was up-regulated over 50-fold in M1 macrophages as compared to unstimulated M0 macrophages (**Fig 3D–3F**). In contrast, CD38 expression was decreased in M2 macrophages. The second candidate M1 marker, Fpr2, was up-regulated over 100-fold during M1 differentiation but down-regulated during M2 differentiation. Finally, Real-Time PCR confirmed that Gpr18 was up-regulated over 35-fold in M1 macrophages. Raw Ct averages for Fpr2, CD38 and Gpr18 in M2 macrophages were >32, 35 and 36, respectively. Overall, Fpr2, CD38 and Gpr18 represent three highly expressed, M1 macrophage-specific markers.

### 3.5. Identification and validation of distinct M2 macrophage markers

**Fig 4:**
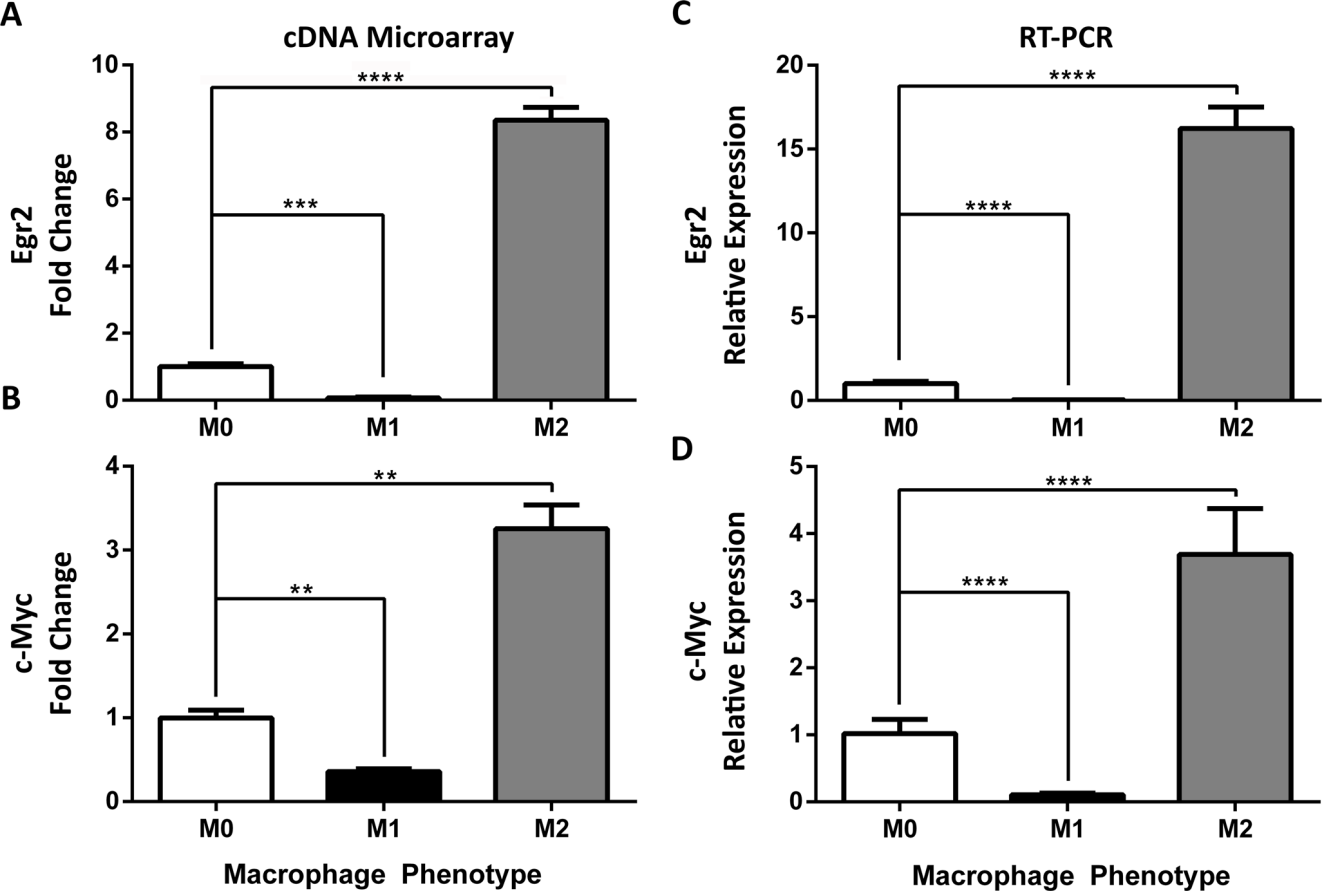
Identification and validation of alternatively activated M2 markers c-Myc and Egr2. Expression of (**A**) Early Growth Response Protein-2 (Egr2) and (**B**) c-Myc was determined using cDNA microarray in BMDMs stimulated for 24 hours in M0, M1, and M2 (n = 2–3 mice, 2 replicates/mouse/condition) conditions. Gene expression is represented as fold change (FC ± SEM) relative to unstimulated M0 condition; multiple comparison corrected t-test. Expression of **(C)** Early Growth Response Protein-2 (Egr2) and **(D) c-**Myc expression was measured via RT-PCR and expressed as mean relative expression (±SD) in M0, M1, and M2 (n = 8, 6 mice, 1–2 replicates/mouse/condition) BMDMs. Gene expression is expressed as FC ± SD of unstimulated M0 condition; ANOVA followed by multiple comparison post-hoc t-test; **p<0.01, ***p<0.001, ****p<0.0001. Data shown are from one experiment representative of two independent experiments.

To identify M2 markers, we focused on the genes highlighted in green in **Fig 2**, which were up-regulated during M2 polarization but down-regulated during M1 polarization. Among those genes, Egr2 was among the most highly up-regulated genes, with over an 8-fold change increase (**Fig 4A**) while its expression decreased more than 10-fold in M1 macrophages. c-Myc is an important TF in the immune system predicted to be linked to Egr2 by Ingenuity Pathway Analysis (**S3 Fig**). Array data showed a 3-fold increase (p≤0.01, **Fig 4B**) in c-Myc in M2 macrophages but a 2-fold decrease in M1 macrophages (**Fig 4B**).

To validate these M2 genes, we performed Taqman RT-PCR on independent datasets. Egr2 expression was increased 16-fold (**Fig 4C**) in M2 macrophages compared to unstimulated M0 macrophages but decreased 26-fold ± 3.5 in M1 macrophages. c-Myc expression was increased 3-fold in M2 macrophages (**Fig 4D**) but decreased 9-fold in M1 macrophages. Importantly, raw Ct averages for Egr2 and c-Myc in M1 macrophages were >37 and 35, respectively, confirming low-level expression in these cells.

### 3.6. CD38/Egr2 flow assay discriminates M1/M2 macrophage phenotypes

**Fig 5:**
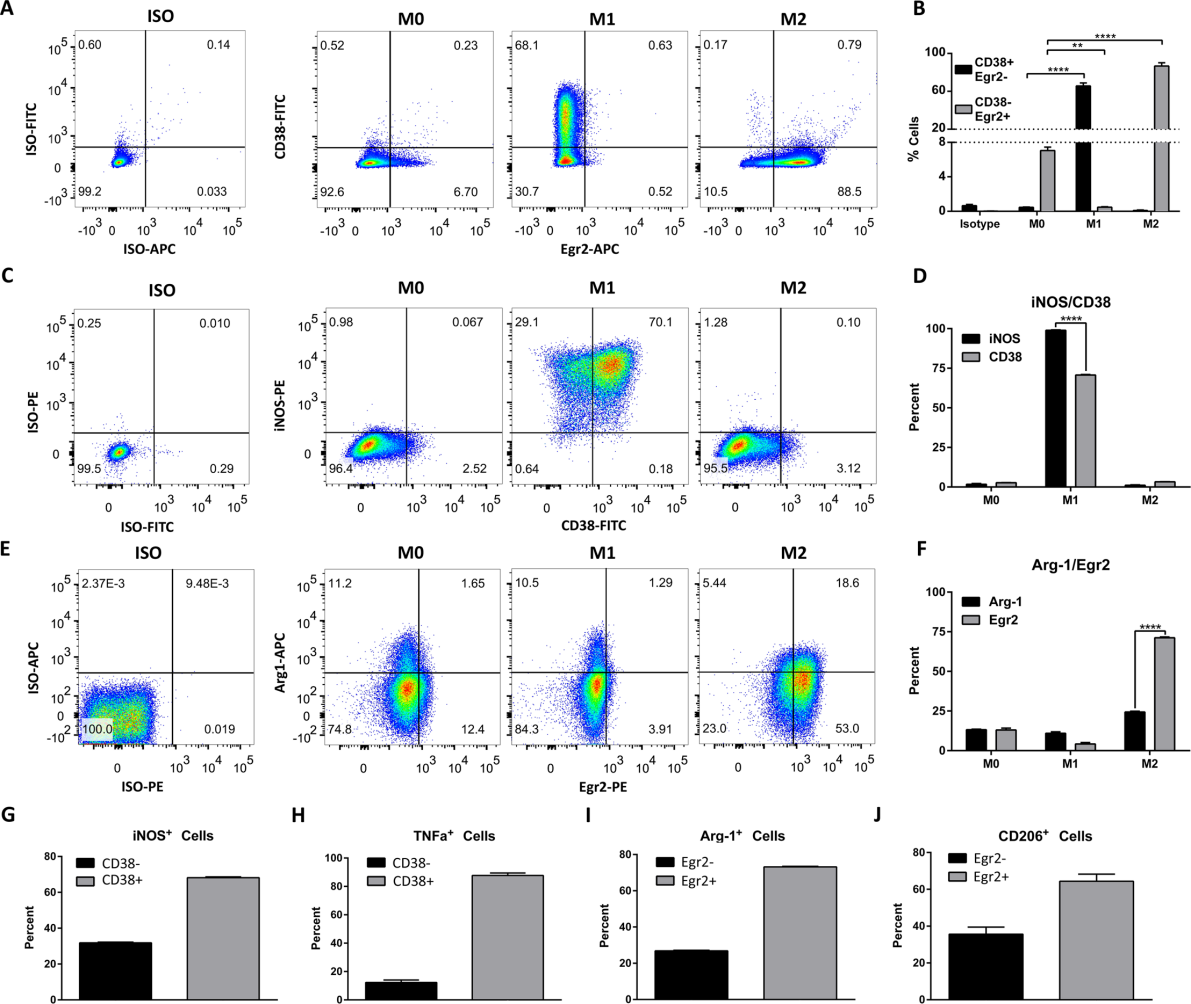
Expression of CD38 and Egr2 protein in M0, M1 and M2 macrophages. Flow cytometry staining of surface CD38 and intracellular Egr2 (**A**), CD38 and iNOS (**C**) or Egr2 and Arginase-1 (**E**) in BMDM 24 hours post-differentiation in M0, M1 and M2 conditions (representative data of three independent experiments). Flow plots correspond to the CD11b gate. **B.** Quantification of **A**, showing the proportion of M0, M1 and M2 macrophages with CD38^+^Egr2^-^ (putative M1) or CD38^-^Egr2^+^ (putative M2) phenotype at 24 hours (n = 3–6 replicates from two independent experiments). **D.** Quantification of **C**, showing the proportion of M0, M1 and M2 macrophages with CD38^+^ or iNOS+ phenotype at 24 hours (n = 3 replicates, representative of three independent experiments). **F.** Quantification of **E**, showing the proportion of M0, M1 and M2 macrophages with Egr2^+^ or Arg-1^+^ phenotype at 24 hours (n = 3 replicates representative of three independent experiments). Percentage of CD38^+^ cells in iNOS^+^ (**G**) or TNF-α^+^ (**H**) BMDM differentiated for 24 hours in M1 conditions. Percentage of Egr2^+^ cells in Arg-1^+^ (**I**) or CD206^+^ (**J**) BMDM differentiated for 24 hrs in M2 conditions. **p<0.01, ****p<0.0001.

To confirm that the above genes are translated into protein and to attempt to use these proteins as markers capable of distinguishing between M1 and M2 macrophage populations, we designed a flow cytometry panel to detect membrane CD38 and intracellular Egr2. BMDMs were differentiated using M0, M1 or M2 stimulation conditions for 24 hours then were stained with antibodies against CD11b, surface CD38 and intracellular Egr2 or isotype controls. We expected M1 cells to display a CD38^+^Egr2^-^ phenotype and M2 cells to have a CD38^-^Egr2^+^ phenotype. Indeed, 65.6% ± 3 (SD) of cells cultured in M1 conditions expressed surface CD38 and were negative for intracellular Egr2 staining, whereas <1% of cells cultured in M0 and M2 conditions were CD38^+^Egr2^-^ (**Fig 5A**, quantified in **Fig 5B**). Conversely, 86.7% ± 3 (SD) of M2 cells were CD38^-^Egr2^+^ while less than 1% of M1 cells and only 7% of M0 cells displayed this phenotype (**Fig 5A and 5B**). To ascertain that this phenotype was stable over time, we compared the expression of these markers at 24 h and 6 days post-differentiation. At 6 days, 58.0% ± 1 of M1 culture macrophages displayed the CD38^+^Egr2^-^ phenotype (**S4A Fig**) and 81.7% ± 3 of M2 culture macrophages were CD38^-^Egr2^+^ (**S4B Fig**). Importantly, M0 or M2 macrophages did not acquire a CD38^+^Egr2^-^ phenotype over time. Similarly, less than 1% of M1 macrophages were CD38^-^Egr2^+^ at 6 days. In contrast, the CD38^-^Egr2^+^ phenotype reached 24% within the M0 population (**S4B Fig**), consistent with the reported shift to M2 phenotype that may occur during extended *in vitro* conditions [30,31,46]. These data show that while the CD38^+^Egr2^-^ phenotype is associated with M1 macrophages, the M2 CD38^-^Egr2^+^ phenotype is linked to M2 macrophages and that both phenotypes are stable over several days. Comparison to classic markers iNOS and Arginase-1 is shown in **Fig 5C–5J**. While iNOS is expressed in a higher proportion of M1 cells than CD38 (**Fig 5C and 5D**), practically all CD38^+^ cells were iNOS^+^ (**Fig 5C**) and the majority of iNOS^+^ (**Fig 5G**) or TNF-α^+^ (**Fig 5H**) M1 cells co-express CD38. In contrast, while only 24.4 ± 0.5% of M2 macrophages expressed Arginase-1, 71.2 ± 0.4% stained for Egr2 (**Fig 5E and 5F**). Within the relatively small percentage of M2 cells expressing Arginase-1 or CD206^+^, most were positive for Egr2 (**Fig 5I and 5J**). Overall, this indicates that CD38 and Egr2 are M1 and M2 markers, respectively, and that Egr2 is an improved M2 protein marker over Arginase-1 or CD206.

**Fig 6:**
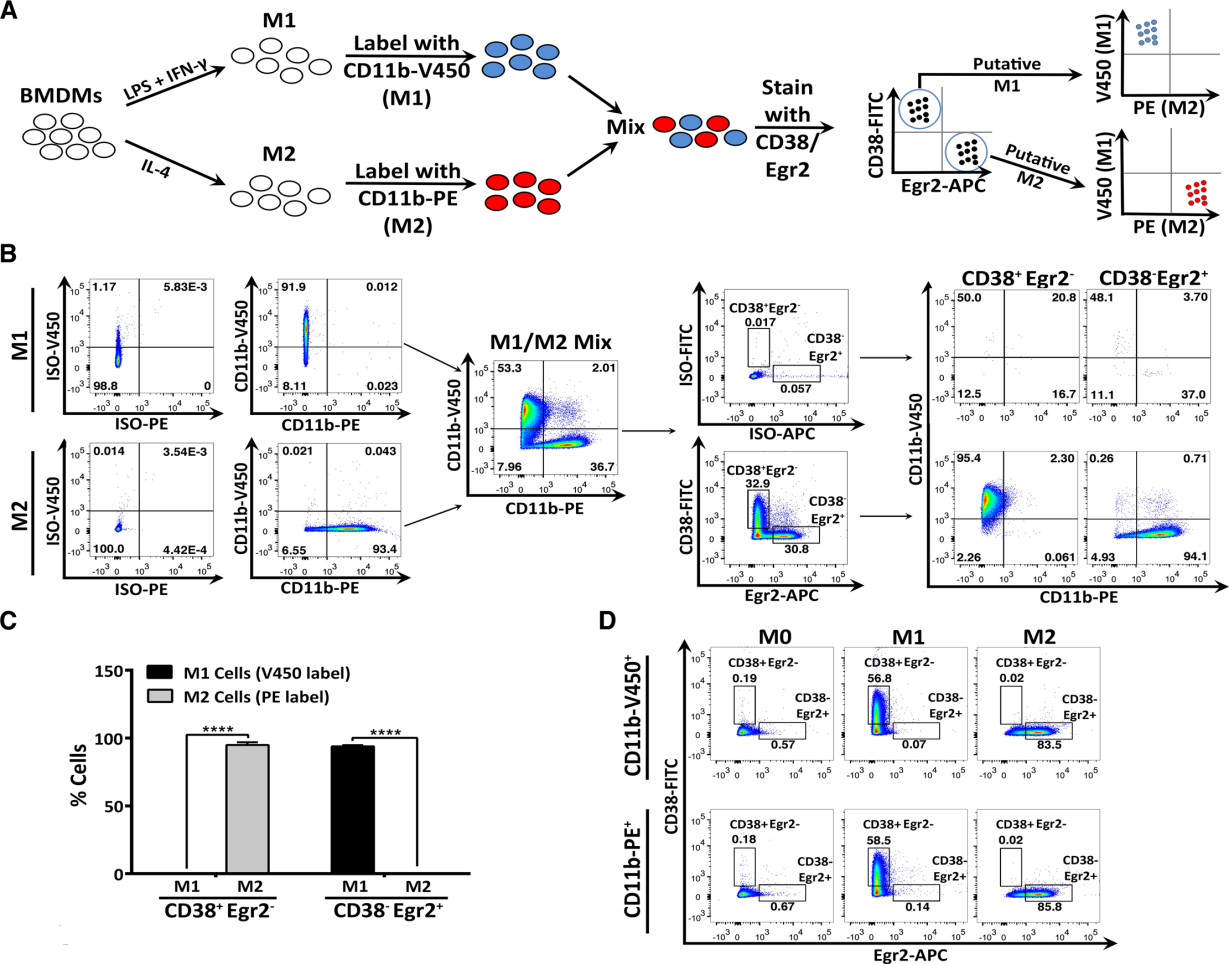
Discrimination of M1 and M2 macrophages via CD38 and Egr2 flow cytometry assay. A. Experimental design. BMDMs were differentiated for 24 hours in M1 conditions and tagged with CD11b-V450. Another set of BMDM was differentiated in M2 conditions and tagged with CD11b-PE. The M1 and M2 populations were then mixed prior to staining with surface CD38 and intracellular Egr2. CD38^+^Egr2^-^ (putative M1) and CD38^-^Egr2^+^ (putative M2) gates were drawn and the percentage of cells in each gate that originated from M1 (V450 tagged) or M2 (PE tagged) cultures was calculated. B. Representative flow cytometry data of CD38 and Egr2 staining in tagged M1 and M2 populations as indicated in A. C. Quantification of the results from B (n = 3, representative of three independent experiments). D. M0, M1 and M2 cells were stained with either CD11b-V450 or CD11b-PE and subsequently stained with CD38 or Egr2 to confirm that CD11b-V450 or -PE tagging did not alter CD38 or Egr2 staining. ****p<0.0001.

To determine if the CD38^+^Egr2^-^ and CD38^-^Egr2^+^ phenotypes could be used to discriminate between M1 and M2 macrophages present in a mixed population, we performed flow cytometry on tagged M1 and M2 cells. **Fig 6A** shows the experimental design in which an M1 culture was V450-tagged via CD11b-V450 staining while an independent M2 culture was PE-tagged via CD11b-PE staining. When mixed, the two-labeled populations provided a heterogeneous macrophage population on which CD38 and Egr2 flow staining could be used to identify CD38^+^Egr2^-^ or CD38^-^Egr2^+^ population gates. We then calculated the proportion of cells within either gate that originated from the M1 (V450-tagged) or M2 (PE-tagged) culture (**Fig 6A**). 94.9 ± 0.8% of CD38^+^Egr2^-^ cells indeed originated from the M1 culture and 93.9 ± 0.3% of CD38^-^Egr2^+^ cells originated from the M2 culture (**Fig 6B, quantified in 6C**). Neither the CD11b-V450 nor -PE tag altered CD38 or Egr2 staining in M0, M1 and M2 cells (**Fig 6D**). These data indicate that CD38 and Egr2 markers can discriminate between M1 and M2 macrophages present in a mixed population.

### 3.7. Mouse endotoxemia promotes a CD38^+^ macrophage population

**Fig 7:**
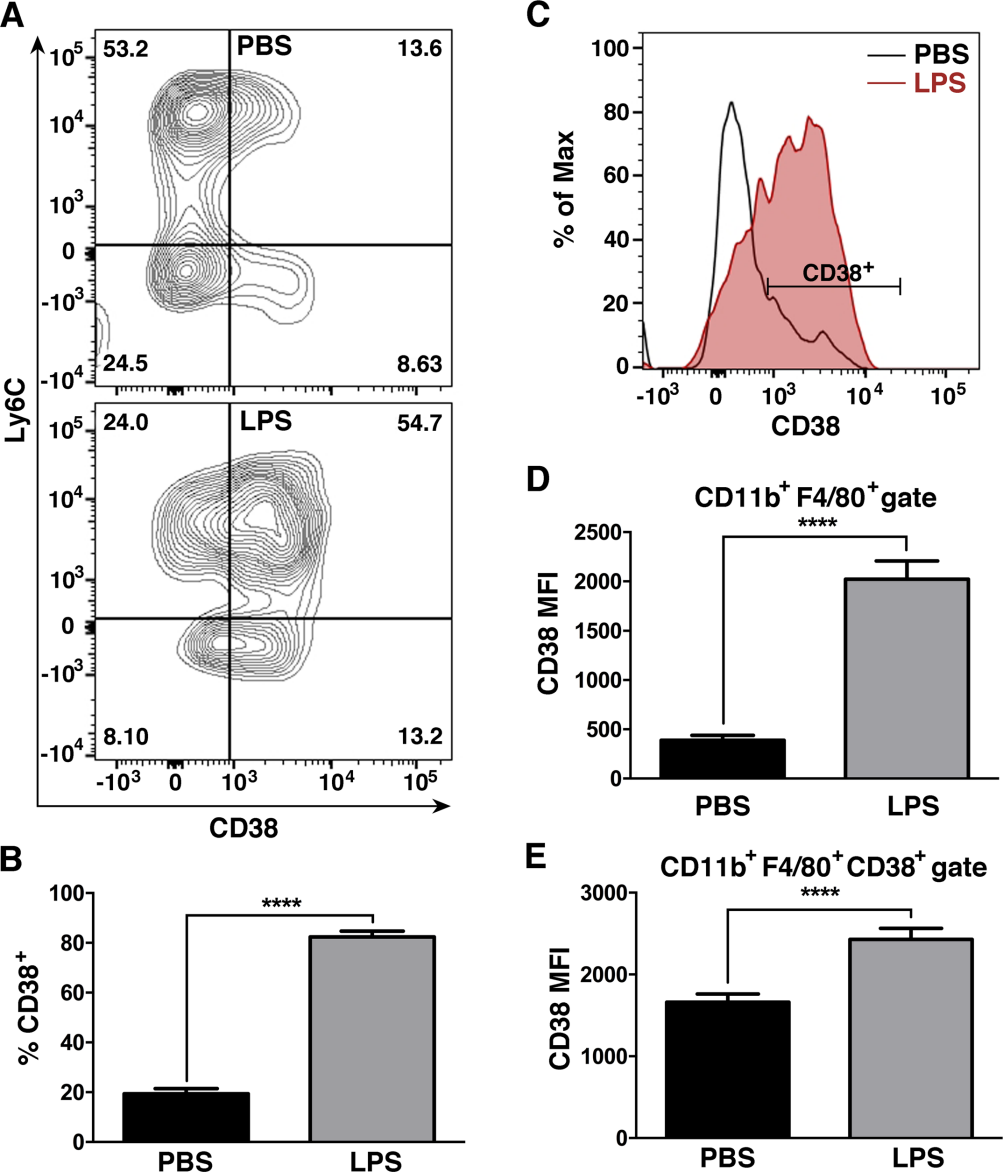
Mouse endotoxemia promotes a CD38^+^ macrophage population. **A.** Representative flow plots of CD38 expression in spleen CD11b^+^F480^+^ macrophages of mice treated for 12 hours with PBS (top) or LPS (bottom). Spleen macrophages were gradient-enriched (see Materials and Methods) and processed for flow cytometry. Flow plots show the CD11b^+^F480^+^ population after gating out dead cells (IndoA^+^) and neutrophils (Ly6G^+^). **B.** Quantification of the results from A (n = 3, representative of three independent experiments). **C.** Histogram plots comparing CD38 expression by Mean Fluorescence Intensity (MFI) in spleen macrophages (IndoA^-^Ly6G^-^CD11b^+^F480^+^ gate) from mice treated with PBS (light blue line) or LPS (shaded thick red line). **D.** Quantification of the results from C (n = 3, representative of three independent experiments). **E.** Comparison of CD38 expression by MFI within the spleen CD38^+^ macrophage population (labeled in **C**) of PBS or LPS-treated mice (n = 3, representative of three independent experiments). ****p<0.0001.

To determine if a similar CD38^+^ inflammatory macrophage population exists *in vivo*, mice were treated with LPS or PBS via *i*.*p*. route for 12 hours. To exclusively evaluate CD38 expression in gradient-enriched spleen macrophages, after excluding neutrophils (Ly6G^+^) and dead (IndoA^+^) cells, our analysis gated CD11b^+^F480^+^ cells. This strategy has been shown to be better than Gr1/F480 staining for the identification of macrophages in the spleen [47]. LPS treatment resulted in a large increase in the percentage of CD38^+^ macrophages (Ly6C^-^) and inflammatory monocytes (Ly6C^+^), compared to PBS controls (**Fig 7A and 7B**). In addition, there was an increase in CD38 mean fluorescence intensity (MFI) in the total CD11b^+^F480^+^ population after LPS-treatment (**Fig 7C and 7D**). While a small CD38^+^ population was detectable in naive (PBS-treated) mice, the population of PBS mice had lower CD38 expression, as measured by a reduced MFI, than the one present in LPS-treated mice (**Fig 7E**). These data indicate that CD38 expression in macrophages is not limited to *in vitro* M1 macrophages, but is also detectable in *in vivo* M1 macrophages after exposure to LPS stimuli.

## Discussion

A better understanding of the molecular pathways and transcriptional programs associated with different macrophage subtypes, as well as reliable markers of macrophage phenotype, are necessary for further progress in the macrophage field. While *in vivo* macrophage characterization is complex and varied over a spectrum of phenotypes [3,9,10], *in vitro* models provide a snapshot of phenotype extremes. Here, we completed a transcriptome analysis of M1 and M2 macrophages followed by validation of candidate M1 and M2 markers. We found that polarizing the phenotype of macrophages using canonical activation stimuli, i.e., LPS+IFN-γ for classical M1 macrophages and IL-4 for alternative M2 macrophage activation, resulted in the expression of shared and distinct, gene expression profiles. Among the genes that were uniquely expressed in either M1 or M2 macrophages, we validated CD38, Fpr2, Gpr18 as M1-specific genes and c-Myc and Egr2 as M2-specific genes.

M1 and M2 macrophages are expected to share a common set of genes involved in basic macrophage functions, such as regulation of macrophage-specific gene transcription, protein synthesis and phagocytosis. We found commonly up-regulated genes including TF Klf4, known to be required for inflammatory monocyte differentiation [48], and previously reported in both M1 [49] and M2 macrophages [50]. Klf4 activity has been linked to Atf4, another TF previously linked solely to LPS-induced TLR signaling [51]. Among the up-regulated TFs, Nfil3, Hivep3 and Bhlhe40 can negatively regulate cytokine gene expression [52–54]. For example, Nfil3 binds to and suppresses IL-12 promoter activity during LPS tolerance. A possible explanation is that some of these commonly up regulated TFs suppress pro-inflammatory gene expression in M2 macrophages, while maintaining pro-inflammatory gene expression in M1 macrophages. The observation that Nfil3 is expressed in LPS-tolerized macrophages would be consistent with this hypothesis [55]. Alternatively, they could control other housekeeping processes common to activated macrophages.

Several membrane proteins were selectively induced in M1 macrophages, including the G-protein coupled receptor proteins Gpr18 and Fpr2, and the ectoenzyme CD38. Fpr2 binds to N-formyl methionyl peptides promoting chemotaxis and the formation of reactive oxygen species (ROS) in phagocytes [56]. Fpr2 appears to have an essential functional role in M1 macrophages since its deficiency promotes an M2 phenotype shift [57]. Fpr2 stimulation by bacterial derived N-formyl methionyl peptides may also induce CD38 mediated signaling pathway in macrophages, as reported to occur in neutrophils [58]. Therefore, these two receptors might converge into an important M1 polarizing signaling mechanism. CD38 is often referred to as an activation marker expressed on lymphocytes, neutrophils and NK cells upon inflammatory conditions [59]. CD38 was linked to LPS networks, but IFN-γ has been also reported to up-regulate CD38 in human monocytes[60]. CD38 promotes signal transduction by catabolizing the formation of the calcium mobilizing messengers cADP-Ribose and ADP-Ribose from NAD^+^, and hence, induces Ca^2+^ influx [61]. CD38 may also enhance M1 macrophage antimicrobial activities by depleting NAD^+^ from extracellular milieu [62]. Consistent with these findings, CD38-deficient mice are reportedly more susceptible to *Streptococcus pneumoniae* [61] and *Mycobacterium tuberculosis* [63] lung infections, where inflammatory macrophages are essential, suggesting possible roles for CD38 during M1 macrophage polarization and function. However, further studies are required to determine the specific role and importance of CD38 in M1 macrophage responses.

With regards to Gpr18, similar to our data, this receptor had previously been found to be up-regulated in inflammatory, but not anti-inflammatory, peritoneal macrophages [64]. However, further work needs to be done to fully determine the specific roles and importance of these markers in M1 macrophage responses.

In M2 macrophages, IL-4 signaling has been linked to expression of the zinc finger TFs, Egr2 and c-Myc [65–66]. Egr2 is a highly conserved TF whose deficiency results in decreased bone mass. This observation is consistent with Egr2 inhibiting osteoclast activity and bone loss [67]. Our IPA analysis showed that Egr2 was linked to c-Myc, a TF involved in cell cycle progression, apoptosis and cellular transformation. In turn, c-Myc has been linked to IRF4 [68], an IRF known to promote M2 gene specificity. In human and tumor associated macrophages, c-Myc has been shown to be required for the alternative activation phenotype, controlling 45% of M2 genes [69]. Although considerable differences are observed between mouse and human M2 biomarkers, its expression in mouse M2 macrophages indicates c-Myc is a conserved TF among the two. Interestingly, c-Myc also directly binds to the conserved M2 receptor Mrc1 gene, regulating its expression [69]. Overall, c-Myc appears to play a crucial role as an M2 TF, and its link to Egr2 warrant additional studies on the role of these TFs in M2 phenotype.

Current analysis of macrophage phenotype by flow cytometry is not optimal due to the intracellular location of most M1/M2 macrophage markers and the low specificity of available antibodies. Arginase-1 is up-regulated not only in the expected M2 macrophages but also in M1 spectrum macrophages [30,31]. In addition, protein expression of Arginase-1 or CD206 is often low for optimal flow cytometry M1/M2 discrimination [32], making the analysis of an individual cell within a macrophage population difficult. Here, we validated surface CD38 as an M1-specific macrophage marker that can be easily detected by flow cytometry. While iNOS staining captured a higher percentage of M1 differentiated cells, virtually all CD38^+^ cells expressed iNOS and most iNOS^+^ or TNF-α^+^ cells co-expressed CD38. The surface localization of CD38 provides an advantage over intracellular iNOS staining, permitting M1 cell sorting for downstream functional assays. In addition, although detection of Egr2 still requires intracellular flow, its detection showed a robust and discriminating expression pattern that will tremendously improve current detection assays for M2 macrophages. Egr2 and CD38 are stably-expressed over time and enable clear discrimination between M1 and M2 macrophages in a mixed population. The availability of a robust M1/M2 discriminating flow assay could have a significant impact on the ability to track macrophage phenotypes during various physiologic and pathogenic conditions or in response to gene mutations or therapeutic interventions. Here, we observed that LPS stimulus strongly increased CD38 expression within the splenic monocytic/macrophage lineage *in vivo*, which demonstrates the relevance of this marker in the characterization of M1 polarized macrophages. Ongoing and future studies are aimed at determining the robustness of this assay in other inflammatory *in vivo* models.

Overall, we have identified a new set of common and distinct M1 and M2 genes that reveal pathways linked to M1 and/or M2 phenotypes. CD38, Gpr18 and Fpr2 were M1-specific while c-Myc and Egr2 were M2-specific genes. A CD38^+^Egr2^-^ phenotype was characteristic of M1 macrophages while M2 macrophages displayed a CD38^-^Egr2^+^ phenotype. Understanding how these genes operate may shed light into how macrophage function and phenotype are induced and/or maintained. In addition, these genes may provide therapeutic targets for macrophage modulation and serve as discriminating biomarkers of macrophage phenotype.

## Supporting Information

S1 Fig Shared M1/M2 macrophage signature. IPA pathway analysis of common (more than 2FC) up-regulated (red) and down-regulated (green) genes in M1 and M2 macrophages compared to M0 macrophages. Only genes identified by IPA analysis to be linked are pictured. Arrows indicate direct interactions. Data shown are from the microarray shown in Figs 1 and 2. Genes up- or down-regulated in transcriptional networks are shown in bold font.
(TIF)

S2 Fig Distinct M1 macrophage signature. IPA pathway analysis of genes more than 2FC up-regulated in M1 while they were more than 2FC down-regulated in M2 macrophages. Only genes identified by IPA analysis to be linked are pictured. Shaded genes represent genes followed up for further validation, as well as the main stimuli, mediators or markers of M1 phenotype. Arrows indicate direct interactions. Dashed arrows represent indirect interactions. Data shown are from the microarray in Figs 1 and 2.
(TIF)

S3 Fig Distinct M2 macrophage signature. IPA pathway analysis of genes more than 2FC up-regulated in M2 and more than 2FC down-regulated in M1 macrophages. Only genes identified by IPA analysis to be linked are pictured. Shaded genes represent genes followed up for further validation, as well as the main stimuli, mediators or markers of M2 phenotype. Arrows indicate direct interactions. Dashed arrows represent indirect interactions. Data shown are from the microarray in Figs 1 and 2.
(TIF)

S4 Fig Stability of the CD38^+^Egr2^-^ (putative M1, A) and CD38^-^Egr2^+^ (putative M2, B) phenotype, determined by flow cytometry staining, from 24 hours to 6 days after differentiation of macrophages in M0, M1 or M2 conditions (n = 3–6 replicates from two independent experiments). (TIF)

S1 Table Genes increased more than 2-FC in both M1 and M2 macrophages. (XLS)

S2 Table Genes decreased more than 2-FC in M1 and M2 macrophages. (XLSX)

S3 Table Categorization of M1 and M2 shared genes. (DOCX)
