# Supplementary figures and images for: Novel Markers to Delineate Murine M1 and M2 Macrophages
Source: PLoS One. 2015 Dec 23;10(12):e0145342. doi: 10.1371/journal.pone.0145342 (PMC4689374; doi:10.1371/journal.pone.0145342)

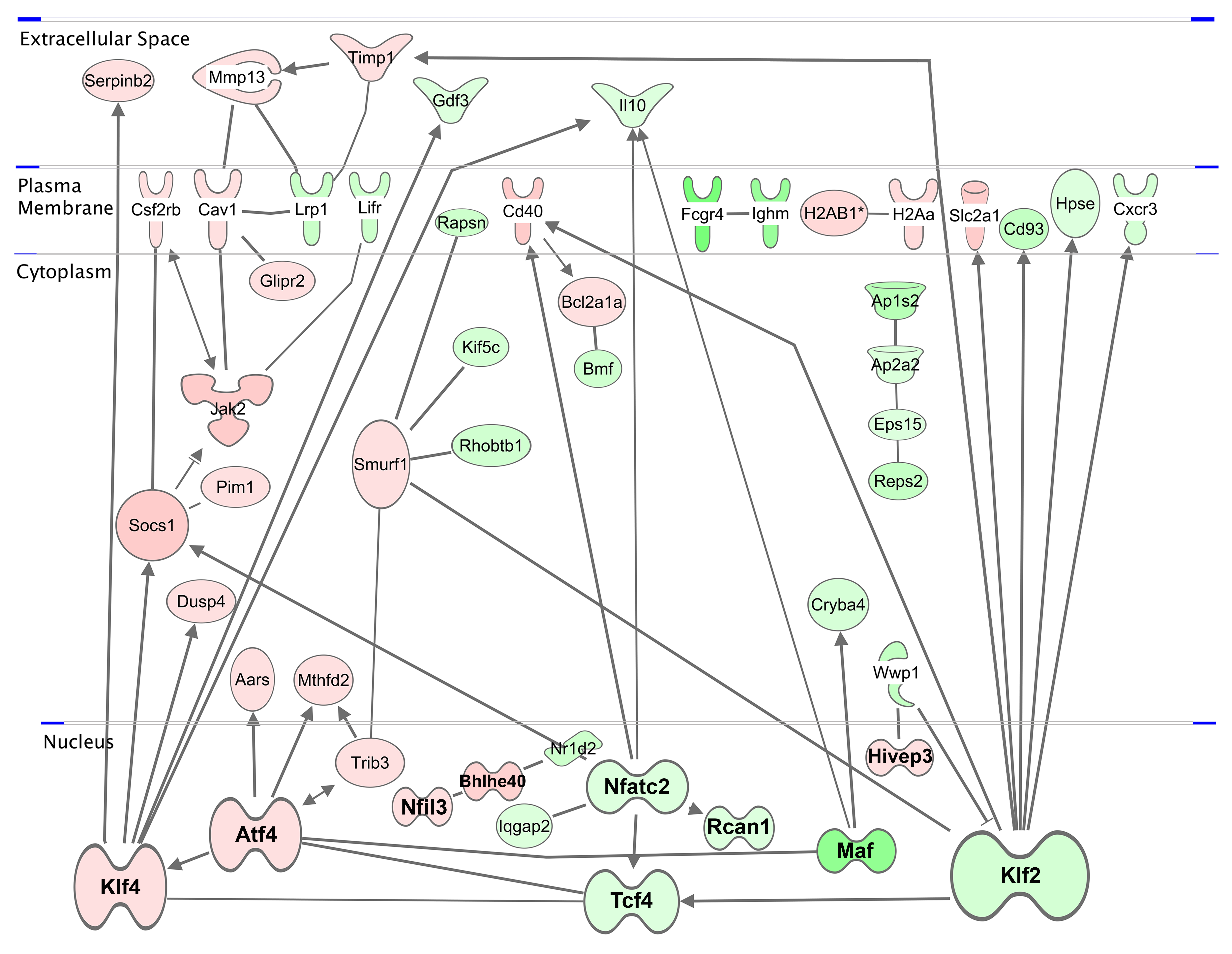

Supplement: S1 Fig — IPA pathway analysis of common (more than 2FC) up-regulated (red) and down-regulated (green) genes in M1 and M2 macrophages compared to M0 macrophages. Only genes identified by IPA analysis to be linked are pictured. Arrows indicate direct interactions. Data shown are from the microarray shown in Figs 1 and 2. Genes up- or down-regulated in transcriptional networks are shown in bold font. (TIF) [file pone.0145342.s001.tif]

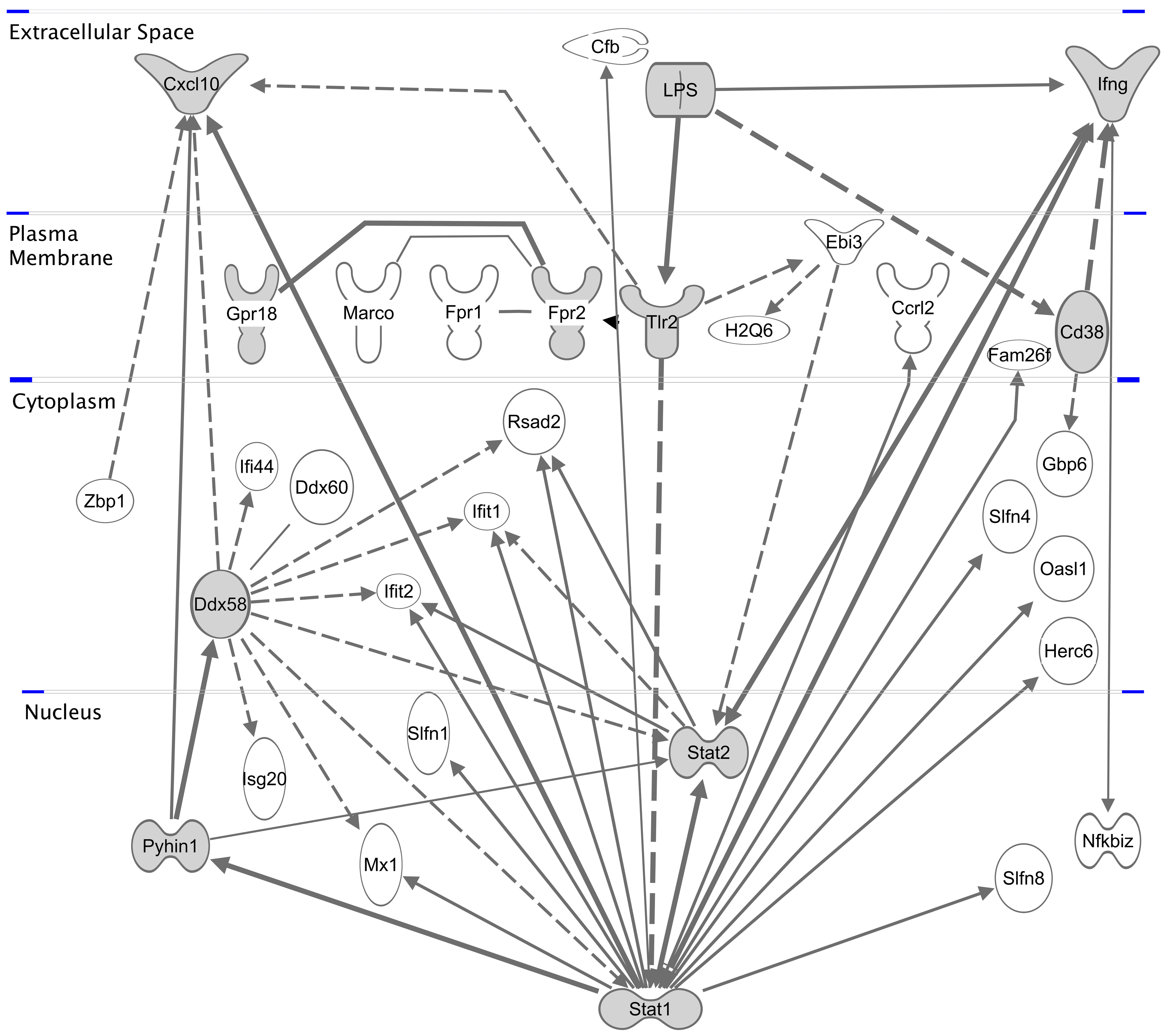

Supplement: S2 Fig — IPA pathway analysis of genes more than 2FC up-regulated in M1 while they were more than 2FC down-regulated in M2 macrophages. Only genes identified by IPA analysis to be linked are pictured. Shaded genes represent genes followed up for further validation, as well as the main stimuli, mediators or markers of M1 phenotype. Arrows indicate direct interactions. Dashed arrows represent indirect interactions. Data shown are from the microarray in Figs 1 and 2. (TIF) [file pone.0145342.s002.tif]

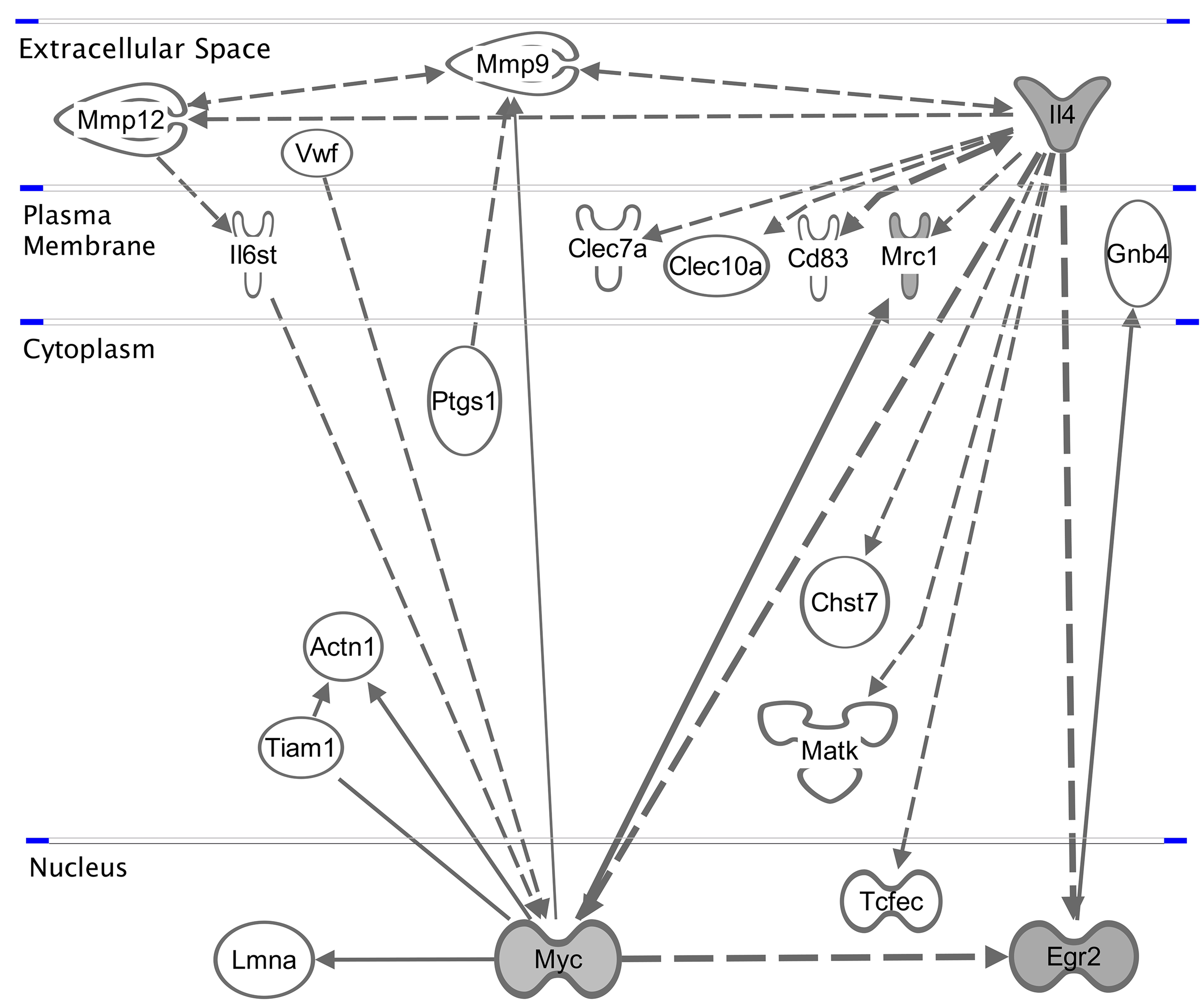

Supplement: S3 Fig — IPA pathway analysis of genes more than 2FC up-regulated in M2 and more than 2FC down-regulated in M1 macrophages. Only genes identified by IPA analysis to be linked are pictured. Shaded genes represent genes followed up for further validation, as well as the main stimuli, mediators or markers of M2 phenotype. Arrows indicate direct interactions. Dashed arrows represent indirect interactions. Data shown are from the microarray in Figs 1 and 2. (TIF) [file pone.0145342.s003.tif]

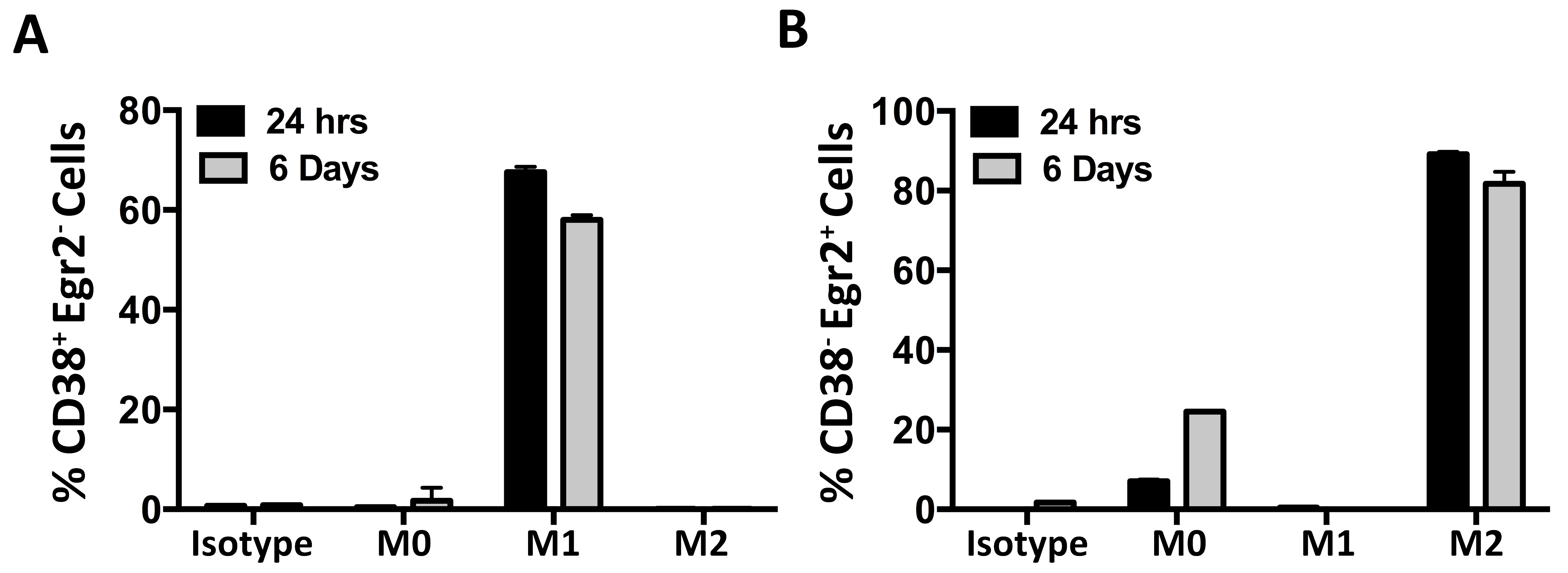

Supplement: S4 Fig — (TIF) [file pone.0145342.s004.tif]
