# Supplementary material for: Novel Markers to Delineate Murine M1 and M2 Macrophages
Source: PLoS One. 2015 Dec 23;10(12):e0145342. doi: 10.1371/journal.pone.0145342 (PMC4689374; doi:10.1371/journal.pone.0145342)
Supplement: S3 Table — (DOCX) [file pone.0145342.s007.docx]

**S3 Table. Categorization of M1 and M2 shared genes.**

|  | **Up** | **Down** |
| --- | --- | --- |
| **Transcription regulator** | Klf4, Bhlhe40, Nfil3, Klf7, Nfkbie, Atf4, Hivep3 | Klf2, Tcf4, Maf, Rcan1, Nfatc2, Nfxl1, Dbp, Sncaip, Zfp36l2 |
| **Enzyme** | Scd2, Rcl1, St3gal1, Nup62-il4i1, Mthfd2, Pde4b, Aars, Alas1, Adhfe1, Smurf1, Pla2g4a, Upp1, Car2, Ch25h, Aars, Aldh1b1 | Cyp27a1, Ada, B3galnt1, Hpgds, Padi4, Zdhhc14, Asph, Rnasel, Wwp1, Rhobtb1, Plscr4, Hpse, B4galt6, Akr1b8, Xylt2 |
| **Transmembrane**  **receptor** | Gpc1, Olr1, Csf2rb, Slamf1, Slamf6, Il1rl2, Fcgr2b, CD40, Cav1, Csfr2b, Timp1 | Fcgrt, Cd28, Itga6, Fcgr4, Klra3, Klra9, Ighm, Lrp1, Evi2a, Cd5l, Lifr |
| **Transporter** | Aqp9, Slc7a2, Slc7a11, Slc2a1, Slco4a1, Syt7, Soat2 | Slc13a3, Slc40a1, Ap2a2, Slc9a9, Atp8a1, Abca9, Slc12a9, Ap1s2, Slc46a3, Folr2 |
| **Kinase** | Jak2, Trib3, Plk3, Pim1, Pim3, | Camk1, Mex3b, Dck |
| **G-protein coupled receptor** | Ptgir, Adora2b, Ptger2, | Gpr162, Ptger4, C5ar1, CXCR3, Gpr155 |
| **Peptidase** | Mmp13, F10, Malt1 | Rnf150, Ctsb |
| **Phosphatase** | Ppap2a, Dusp4 | Ptpn18 |
| **Cytokine** | Ccl22 | Il16, Il10 |
| **Ligand-dependent nuclear receptor** |  | Nr1d2 |
| **Growth factor** |  | Gas6, Gdf3 |
| **Ion Channel** |  | Tpcn1 |
